# A probabilistic approach to learn chromatin architecture and accurate inference of the NF-κB/RelA regulatory network using ChIP-Seq

**DOI:** 10.1093/nar/gkt493

**Published:** 2013-06-14

**Authors:** Jun Yang, Abhishek Mitra, Norbert Dojer, Shuhua Fu, Maga Rowicka, Allan R. Brasier

**Affiliations:** ^1^Department of Internal Medicine, The University of Texas Medical Branch, 301 University Boulevard, Galveston, TX 77555-1060, USA, ^2^Department of Biochemistry and Molecular Biology, The University of Texas Medical Branch, 301 University Boulevard, Galveston, TX 77555-1060, USA, ^3^Institute for Translational Sciences, The University of Texas Medical Branch, 301 University Boulevard, Galveston, TX 77555-1060, USA, ^4^Institute of Informatics, University of Warsaw, Banacha 2, 02-097, Warsaw, Poland and ^5^Sealy Center for Molecular Medicine, The University of Texas Medical Branch, 301 University Boulevard, Galveston, TX 77555-1060, USA

## Abstract

Using nuclear factor-κB (NF-κB) ChIP-Seq data, we present a framework for iterative learning of regulatory networks. For every possible transcription factor-binding site (TFBS)-putatively regulated gene pair, the relative distance and orientation are calculated to learn which TFBSs are most likely to regulate a given gene. Weighted TFBS contributions to putative gene regulation are integrated to derive an NF-κB gene network. A *d*e *novo* motif enrichment analysis uncovers secondary TFBSs (AP1, SP1) at characteristic distances from NF-κB/RelA TFBSs. Comparison with experimental ENCODE ChIP-Seq data indicates that experimental TFBSs highly correlate with predicted sites. We observe that RelA-SP1-enriched promoters have distinct expression profiles from that of RelA-AP1 and are enriched in introns, CpG islands and DNase accessible sites. Sixteen novel NF-κB/RelA-regulated genes and TFBSs were experimentally validated, including TANK, a negative feedback gene whose expression is NF-κB/RelA dependent and requires a functional interaction with the AP1 TFBSs. Our probabilistic method yields more accurate NF-κB/RelA-regulated networks than a traditional, distance-based approach, confirmed by both analysis of gene expression and increased informativity of Genome Ontology annotations. Our analysis provides new insights into how co-occurring TFBSs and local chromatin context orchestrate activation of NF-κB/RelA sub-pathways differing in biological function and temporal expression patterns.

## INTRODUCTION

**Figure 1.**
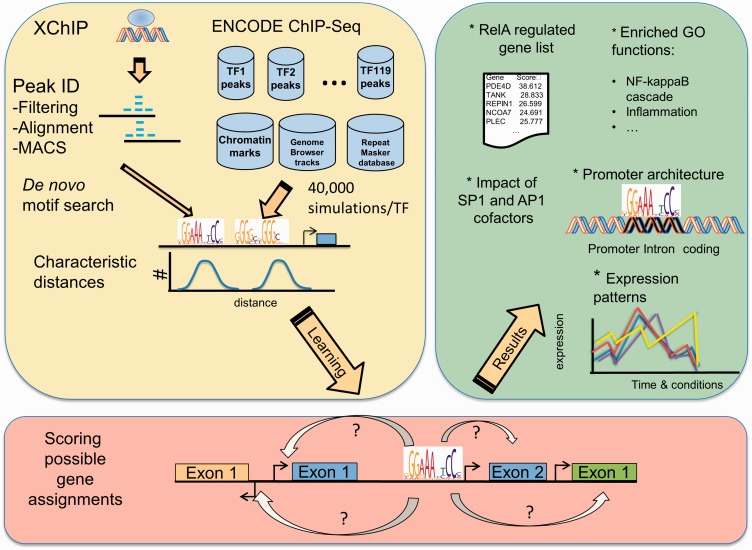
Workflow of ChIP-Seq/RNA-Seq analysis pipeline. NF-κB/RelA XChIP data were processed and peak identification using model-based approaches. High-confidence NF-kB/RelA-binding peaks were subjected to exhaustive *de novo* motif searching, and compared with transcription factors (TFs) and chromatin modification data within the ENCODE data set. A probabilistic method was developed to assign NF-κB/RelA motifs to the most likely regulated gene. The NF-κB/RelA gene network was in further analyzed for characteristic distances between NF-κB/RelA and co-occurring *cis* elements, mapped to structural regions of candidate genes and expression profiles validated by RNA-Seq.

Systematic elucidation of gene regulatory networks is a major focus of systems biology. Recent rapid advances in deep sequencing and resulting availability of massive publicly available chromatin immunoprecipitation (ChIP)-Seq and RNA-Seq data sets [e.g. through the ENCODE consortium (1,2)] make developing computational methods for unsupervised, high-level analysis of such data even more important and timely. Although there has been significant progress in the methods for primary statistical analysis of ChIP-Seq data (3), much less attention has been devoted to accurate assigning of ChIP-Seq transcription factor-binding sites (TFBSs) to genes they putatively regulate. Typically, it is assumed that a TFBS regulates gene whose transcription start site (TSS) is closest to it, often with an added condition that the distance between a TFBS and a gene TSS has to be shorter than an arbitrary threshold (4,5). Such methods are far from optimal, as was recently documented by Gerstein and colleagues (6). Gerstein and colleagues (6) also proposed a more sophisticated approach to associate TFBS with a putatively regulated gene, relying on comparing read pattern and distance to the closest TSS with a typical read pattern and distance for a considered transcription factor (TF). This approach has been used in analysis of the recent ENCODE large-scale ChIP-Seq data (2). However, in the discussion of the results of this massive ENCODE study, developing better methods for associating TFBSs with its regulated gene was named as one of areas needing most improvement as well as the one most crucial for validity of the overall results (2). Here, we present such an improved approach. Unlike previous work (6), our method also includes biological context (e.g. intron location), does not have any distance cut-off and instead of using a simplistic read pattern overlap followed by *Z*-score calculation approach, our method uses leading software, MACS, for highly accurate detection of TFBSs (Figure 1). Our approach for uncovering regulatory networks is probability-based and is designed to more fully exploit information from ChIP-Seq experiments. As a proof-of-concept, we apply our method to reconstruct with high accuracy the regulatory network of the NF-κB/RelA TF.

NF-κB is a family of heterodimeric transcription factors that are inactivated in the cytoplasm of resting cells by reversible association with the IκB family of inhibitors, a family of ankyrin repeat containing proteins that include IκB-α, -β, -γ, or unprocessed forms of cytoplasmic NF-κB-1 and -2 (7,8). NF-κB is the major arm of the innate inflammatory response that induces genetic programs controlling inflammation and anti-apoptosis (9–12). NF-κB activation is controlled by distinct regulatory pathways, termed the ‘canonical’, ‘non-canonical’ and ‘cross-talk’ pathways (13). The most intensively studied of these, the canonical NF-κB pathway, controls the coupled nuclear translocation–transactivation of the potent transactivating 65 kDa NF-κB/RelA subunit (13,14) in response to extracellular stimulation by monokines [tumor necrosis factor alpha (TNFα)] released by activated macrophages. TNF binding induces aggregation of TNF receptors, recruitment of the rate-limiting IκB kinase (IKK) and downstream phosphorylation of the IκBα inhibitor, making it a substrate for proteolysis through the 26S proteasome and calpain pathways (15,16). Liberated from its IκBα inhibitor, the heterodimeric RelA•NF-κB1 TF is activated by serine phosphorylation (17,18) and rapidly enters the nucleus.

Within the nucleus, NF-κB/RelA binds to near palindromic 11-nt sequence motifs (19). Here, NF-κB induces gene expression by promoter-specific recruitment of histone-modifying co-activators (p300/CBP, Src 1 and NCoR) to produce chromatin remodeling (20), pre-initiation complex assembly, resulting in transcriptional initiation enhancement (21) and induction of translational elongation (14,22). mRNA profiling studies conducted in the presence of NF-κB inhibitors have identified that NF-κB/RelA regulates several hundred genes important in the inflammatory and anti-viral response, whose expression patterns are temporally controlled in a stimulus-specific manner (11,12). This stimulus-selective regulation is due, in part, to NF-κB/RelA interactions with adjacent *cis*-regulatory modules. Although RSV-inducible IL-8 expression is dependent on NF-κB/RelA interaction with an upstream IRF3-binding site, TNF-inducible IL-8 expression is dependent on NF-κB/RelA interaction with an AP1-binding site (23,24).

To further understand the modulation of NF-κB/RelA activity, an in-depth, genome-wide analysis of its cistrome is needed. Current methods of uncovering regulatory networks are limited to TFBS near the TSS (25,26), even though it is well known that majority of experimentally found TFBSs are outside of traditionally defined promoters. For example, only 7% of NF-κB/RelA TFSBs lie in gene promoters (27). Moreover, typically only genes likely to be regulated by strong TFBSs are listed, instead of integrating contributions of all TFBSs in a gene vicinity into the chance for a given gene to be regulated, as we propose. As a result of this typical approach, many regulated genes still remain undiscovered, as illustrated by TANK, previously unknown NF-κB-regulated gene, which was scored as second most likely NF-κB-regulated gene by our method.

In this study, we demonstrate a systematic analysis of the NF-κB/RelA cistrome in response to its activation in airway epithelial cells. Using two step chromatin immunoprecipitation (XChIP)-Seq of control and TNF activated epithelial cells, a data set of 20 733 high-quality binding sites was obtained. *De novo* motif enrichment analysis of NF-κB/RelA-interacting chromatin shows enrichment for either AP1- or SP1-binding motifs and the presence of a flexible DNA break point domain that occur within characteristic distances of the NF-κB/RelA sites. Notably, RelA-AP1- and RelA-SP1-enriched motifs segregate into two populations, where RelA-SP1-enriched promoters have more rapid onset of mRNA expression profiles, are enriched in intronic locations, CpG islands, open chromatin configuration (DNase accessible sites and phosphorylated Pol II binding) than are RelA-AP1 genes. We conclude that our method for motif–gene regulatory relationship and *de novo* motif search yields comprehensive identification of NF-κB-regulated genes and insights into how co-occurring transcription factors and local chromatin context possibly orchestrate activation of NF-κB sub-pathways.

## MATERIALS AND METHODS

### Cell culture

Human A549 pulmonary type II epithelial cells (American Type Culture Collection, ATCC) were grown in F12K media with 10% fetal bovine serum (FBS), penicillin (100 U/ml) and streptomycin (100 µg/ml) at 37°C in a 5% CO_2_ incubator. HeLa^tTA/FLAG-IκBα Mut^, Tet-transactivator (tTA)-expressing HeLa cells stably transfected with a Tet operator controlled non-degradable IκBα (IκBα Ser32Ala/Ser36Ala) plasmid, were cultured as described previously (10,11). HEK293FT cells (Invitrogen) were maintained in Dulbecco’s modified Eagle’s medium containing 10% FBS, 1% penicillin/streptomycin and 1% glutamine at 37°C in 5% CO_2_. Where indicated cells were stimulated with TNFα (Pepro Tech), 30 ng/ml.

### XChIP

A549 cells at a density of 4–6 × 10^6 ^per 100-mm dish were washed twice with phosphate-buffered saline. Protein–protein cross-linking was first performed with disuccinimidyl glutarate (Pierce) followed by protein–DNA cross-linking with formaldehyde as previously described (28). Equal amounts of sheared chromatin were immunoprecipitated overnight at 4°C with 4 µg indicated Ab in ChIP dilution buffer (28). Immunoprecipitates were collected with 40 μl protein-A magnetic beads (Dynal Inc), washed and eluted in 250 µl elution buffer for 15 min at room temperature (28).

Sonication was adjusted in sodium dodecyl sulfate to produce fragmented chromatin of 200–500-bp size (29). Samples were digested with RNase A treatment before proteinase K digestion to reduce background (30). The ChIP-enriched fragments were then end-repaired, poly A-tails added using 5′–3′ exonuclease (exo-) Klenow enzyme and ligated to sequencing adapters as directed by the manufacturer (New England Biolabs). The library was size selected using E-Gel® SizeSelect™ 2% Agarose (Invitrogen), and 350-bp fragments were eluted and amplified by 15–18 cycles of polymerase chain reaction (PCR). Validation and quantification was performed by quantitative genomic PCR (Q-gPCR)-based method (29), and the library was analyzed for size distribution by fractionation using an Agilent Technologies 2100 Bioanalyzer. The resultant sample libraries were subjected to single-end sequencing on an Illumina Genome Analyzer II for 40 cycles.

### Data analysis

#### Quality analysis and filtering

To ensure best quality of the data, we have started the analysis from the fastq files (containing quality scores for each base called). As a first step, we used our in-house ‘Instant Sequencing’ software to determine the quality of each read and then trim reads at the location where one base was called with confidence <99% (corresponding to phred score ≥20). Only reads that were at least 35-bp long were preserved in a corresponding fasta file. At the end of this step, we obtained two fasta files, one each for TNF treatment at 0 min (TNF-0) and the TNF treatment at 30 min (TNF-30). The TNF-0 sample and the TNF-30 sample contained 17 862 626 and 12 624 879 quality filtered reads, respectively (Supplementary Table S1).

#### Read mapping

We used bowtie v0.12 software (31) to map quality filtered reads from TNF-0 and TNF-30 fasta files to the latest assembly of the human genome, GRCh37/hg19. We used the most conservative parameters for bowtie (zero mismatches, uniquely mappable reads). After the alignment, 77% of reads were uniquely mapped without mismatches for TNF-0 sample and 81% for the TNF-30-treated sample (Supplementary Table S1).

#### Peak calling

RelA ChIP-Seq peaks were called using MACS v0.14 (3). This version of MACS has the ability to accurately call peaks even if the number of mapped reads differs substantially between the control and treatment samples. We used standard MACS parameters for calling peaks in human data, with TNF-0 sample as the control and TNF-30 sample as the treatment. In total, 20 733 peaks were called. Instant Sequencing parses the output file produced by MACS for further analysis. The important parameters that are needed by our tool include the peak location, the significance score of each peak and the location of peak summit. To confirm reproducibility, a second RelA ChIP-Seq replicate was performed and analyzed. A comparison of peak location showed a highly statistically significant peak overlap (*P* < 0.0001).

#### Regulated gene assignment based only on TFBSs located within gene promoters

Instant Sequencing was then used to find TSSs of known human genes proximal to thus obtained ChIP-Seq peaks, using annotations from the GENCODE V12 database. Peaks whose summits were within a distance of 2-kb upstream or downstream of a known TSS of a gene were identified. For each ChIP-Seq peak, a list of TSSs ranked by the increasing order of their distances from the summit of a ChIP-Seq peak was generated. Next, the gene with its TSSs closest to the summit of a peak was identified, and that gene was reported, along with the MACS significance score of that peak. When multiple peaks are proximal to the same gene, the significance scores (which are logarithm of probabilities) for each peak are added together, and the sum is reported as a single score for that gene. Finally, a list of proximal genes ordered by their computed significance scores is generated for finding enriched Gene Ontology (GO) functions.

#### Motif finding

For each peak, the Instant Sequencing extracts from the genome the 500-bp long fasta sequence centered on its summit. This set of fasta fragments is used to perform *de novo* motif search. Sequence motifs were discovered using parallelized MEME v4.4 with –zoops option, motif width ranging from 6 to 25 nt and searching for 10 best motifs. Depending on parameters, the canonical NF-κB/RelA-binding motif was found at between 63 and 100% of the top 20% ChIP-Seq peaks. For further analysis, motifs of 12-nt width were selected because at this width, the canonical NF-κB/RelA-binding motif exhibits highest information content, and the highest number of known or genuine motifs were present among top 10 hits.

#### In silico validation of motifs found enriched with MEME

We devised a statistical approach to determine whether a motif reported by MEME is truly enriched in the genome. The procedure will be described in full elsewhere. Briefly, at each position in the genome, we calculate the likelihood that the observed motif comes from a given motif (as described by position weight matrix) and that it arises randomly. Then, assuming there is no more than one true binding site within a 500-bp fasta fragment derived from the peak (or same length fragment of a genome), we calculate probability for the highest scoring motif using Bonferroni multiple hypothesis testing correction. Then we calculated the expected number of motif occurrences both within peaks and in the whole genome and use hypergeometric probability distribution to determine whether motif occurrences are significantly enriched or depleted.

#### Intra-motif distances

For each gene, we select only one motif of each kind, the one with the best *P*-value. Thereafter, pairwise distances between the motifs assigned to the gene are generated for all the regulated genes. For each pair of motifs, a histogram is generated from the intra-motif distances (≤500 bp). For this study, we have shown histograms depicting typical distribution of distances between NF-κB/RelA motif as the datum and AP1, SP1, break point motifs. The plots indicate inter-motif distance preferences for studied pairs of TFs.

#### Peak-TSS distance histogram

A list of all TSSs was obtained by downloading the human TSS track, published by Switchgear Genomics and available from the USCS Genome Browser (32). Instant Sequencing calculates and reports the distances from the center of ChIP peaks to the best TSS of a given gene. The histogram of these distances is plotted.

#### Analysis of Gene Ontology functions

Analysis of enriched and depleted GO functions was performed using Informativity tools (http://informativity.utmb.edu) (Supplementary webpage, file D). Pathway analysis was performed using ingenuity pathway analysis (http://www.ingenuity.com).

#### Analysis of interactions with Alu and other repeats

Repeat Masker track was downloaded from the UCSC genome browser. Instant Sequencing performed calculation of motif-repeat distances, as described earlier in the text. For enrichment and depletion analysis, we created feature data sets from RepeatMasker (33) and from the CpG Islands and Open Chromatin DNaseI tracks of UCSC Genome Browser (32). As repetitive regions tend to be underrepresented among uniquely mapped reads, in this analysis, we considered only mappable regions. To determine whether a given genomic feature is enriched in NF-κB/RelA peaks, we computed the proportion of feature nucleotides in NF-κB/RelA peaks, as well as their proportion in the whole genome. Next, we performed 100 000 random assignments of NF-κB/RelA peaks to non-overlapping genome locations of respective mappable lengths. Based on these assignments, we calculated the empirical distribution of the feature proportion in NF-κB/RelA peaks under the null hypothesis that the feature and NF-κB/RelA peaks are independently distributed in the human genome. We used this distribution to estimate the *P*-values for the feature enrichment and depletion inside NF-κB/RelA peaks. Note that as we performed 100 000 assignments, the best achievable *P*-value was 10^−5^.

#### Comparison with the ENCODE 119 TF ChIP-Seq data set

The ENCODE ChIP-Seq data for 119 human TFs (1,2,27) was used. As TFBSs are highly enriched in ∼8% of the human genome (1), analysis of enrichment of other TFBSs in NF-κB/RelA peaks yielded highly significant enrichment for almost all considered TFs, when compared with random localizations in the human genome. Therefore, we decided to analyze, for each TF, the deviation of normalized overlaps of its peaks with NF-κB/RelA peaks from the normalized overlaps for all TFBSs. Specifically, for each TF peak, we computed its overlap with NF-κB/RelA peaks and divided it by the length of the TF peak. Then we averaged results for each TF. Next, we randomly shuffled TF labels of peaks and re-calculated average normalized overlaps for all TFs. This procedure was repeated 40 000 times to estimate statistical significance of enrichment and depletion with resolution of 0.000025.

#### Estimating odds for a TFBS to regulate a given gene

**Figure 2.**
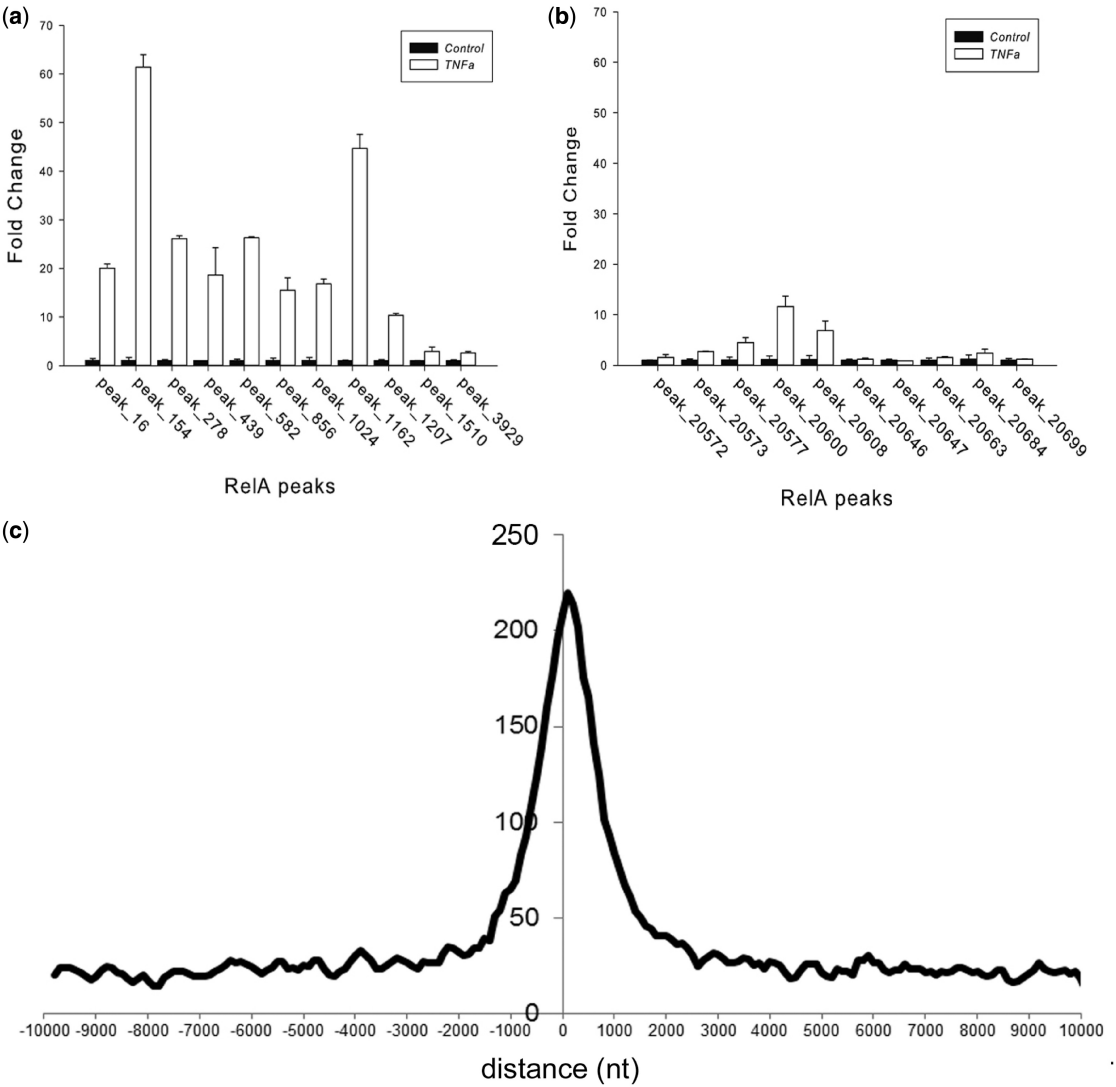
Q-gPCR target validation. (**A**) Eleven novel MACS peaks with the top 20% significance scores were selected for validation (scores >200, Supplementary Table S2). A549 cells were stimulated in the absence or presence of TNF (30 ng/ml, 30 min) and subjected to XChIP. Sheared chromatin was immunoprecipitated with anti-RelA Ab. Shown are Q-gPCR assays for expressed as fold change relative to unstimulated cells for each peak. Primer sequences are given in Supplementary Table S2. A strong (>2-fold) TNF inducible enrichment was observed for each peak analyzed. Here, 11 of 11 MACS peaks were validated successfully (false-positive rate is estimated <6%). (**B**) Ten novel MACS peaks with significance scores <20% were selected for validation (scores <50, Supplementary Table S2). Chromatin from control or TNF stimulated subjected to XChIP, immunoprecipitated with anti-RelA Ab and Q-gPCR performed for each indicated MACS peak. Five peaks show enrichment of >2-fold relative to control chromatin. Primer sequences are given in Supplementary Table S2. (**C**) Distribution of NF-κB/RelA peaks relative to TSS. Shown is a histogram of the distances of NF-κB/RelA summits to the primary TSS of known genes. Based on this histogram, we used a distance of ±2 kb from the peak summit to the TSS as a threshold for traditional method of defining regulated genes.

After initial observation that NF-κB/RelA peaks are enriched within 2-kb upstream or downstream of TSSs (Figure 2c), we have divided all possible TFBS locations relative to the TSS into 16 categories, based on both distance from TSS, an upstream or a downstream position and an intronic, exonic or intragenic location (Table 1). For each category, we computed the enrichment of NF-κB/RelA ChIP-Seq peaks in such a configuration versus random positioning of NF-κB/RelA peaks in the human genome (Table 1). Thus, derived odds were later used to decide which gene is most likely to be regulated by a given NF-κB/RelA motif and to resolve common issue of two TSSs occurring in close proximity to a NF-κB/RelA TFBS.

**Table 1.** Odds of gene regulation based on distance and location of NF-κB/RelA ChIP-Seq peaks with respect to the gene TSS

Distance Bins	Structural Location	Average odds of regulation	SD ±
0 to 2 k	upstream	6.73	0.49
−2 k to 0	intron	4.37	0.21
−2 k to 0	exon	2.19	0.03
−2 k to 0	downstream and outside transcript	2.11	0.52
−25 k to −2 k	downstream and outside transcript	2.01	0.06
2 k to 25 k	upstream	1.73	0.18
−25 k to −2 k	intron	1.39	0.04
−1 Mb to −25 k	downstream and outside transcript	1.21	0
25 k to 1 Mb	upstream	1.07	0
−1 Mb to −25 k	intron	0.85	0.05
−25 k to −2 k	exon	0.84	0.11
−1 Mb and beyond	downstream and outside transcript	0.34	0.01
1 Mb and beyond	upstream	0.32	0.07
−1 Mb and beyond	intron	0.18	0.25
−1 Mb to −25 k	exon	N/A	N/A
−1 Mb and beyond	exon	N/A	N/A

The odds of regulation are computed as ratio of frequencies of observing a given location in NF-κB/RelA ChIP-Seq peaks divided by frequencies of observing such location if peaks were randomly placed in the genome, separately for the positive and negative strands. Then, average and standard deviation was computed using data from both strands. Note that as our data includes both false positives and false negatives, the reported ratios are likely conservative estimates showing minimum possible values. ‘N/A’ indicates the cases where there was not enough data to allow estimating odds of regulation.

#### Estimating likelihood of a gene being NF-κB/RelA regulated

Finally, we scored all genes according to their likelihood of being regulated by NF-κB/RelA. This scoring was done by multiplying probabilities of a given gene being NF-κB/RelA regulated based on all NF-κB/RelA TFBSs in the vicinity of its TSS, for which the given gene was considered the most likely target. The full list is provided on the Supplementary webpage (http://nfkb.utmb.edu, file B).

#### Validation using quantitative real-time genomic PCR

Gene enrichment in XChIP was determined by quantitative real-time genomic PCR (Q-gPCR) as previously described (29) using region-specific PCR primers. The fold change of DNA in each immunoprecipitate was determined by normalizing the absolute amount to input DNA reference and calculating the fold change relative to that amount in unstimulated cells. Primers used for assay of TANK promoter enrichment are forward, 5′-GAAGTCAGAGCTACAGTCA-3′, reverse, 5′-CCAATCATGGCACGATAC-3′.

#### Validation using quantitative Real-Time PCR

For gene expression analysis, 1 µg of RNA was reverse transcribed using Super Script III in a 20 µl of reaction mixture (11). One microliter of cDNA product was diluted 1:2, and 2 µl of diluted product was amplified in 20 µl of reaction mixture containing 10 µl of SYBR Green Supermix (Bio-Rad) and 0.4 µM each of forward and reverse gene-specific primers. The reaction mixtures were aliquoted into Bio-Rad 96-well clear PCR plate, and the plate was sealed by Bio-Rad Microseal B film before putting into PCR machine. The plates were denatured for 90 s at 95°C and then subjected to 40 cycles of 15 s at 94°C, 60 s at 60°C and 1 min at 72°C in an iCycler (BioRad). PCR products were subjected to melting curve analysis to assure that a single amplification product was produced.

Quantification of relative changes in gene expression was calculated using the ΔΔCt method. In brief, the ΔCt value was calculated (normalized to GAPDH) for each sample by the following formula: ΔCt = [Ct (target gene) − Ct (GAPDH)]. Next, the ΔΔCt was calculated by the formula: ΔΔCt = [ΔCt (experimental sample) − ΔCt (control sample)]. Finally, the fold change (FC) between experimental sample and control sample was calculated using the formula FC = 2^(−ΔΔCt). Primers used for quantification of TANK mRNA by quantitative real-time PCR (Q-RT-PCR) are forward, 5′-CAACTGTGTAACTGTCTTCATCT-3′; reverse, 5′-CAGGTCTCTTCTCCAGGTT-3′.

#### shRNA knockdown analysis

Lentiviral AP1 shRNA constructs were purchased from Sigma-Aldrich. The sequences of shRNA oligos targeting the c-Jun gene (NM_002228) are CCGGTAGTACTCCTTAAGAACACAACTCGAGTTGTGTTCTTAAGGAGTACTATTTTTG (TRCN0000010366), CCGGCGGACCTTATGGCTACAGTAACTCGAGTTACTGTA GCCATAAGGTCCGTTTTTG (TRCN0000039589), CCGGTGGGTGCCAACTCA TGCTAACCTCGAGGTTAGCATGAGTTGGCACCCATTTTTG (TRCN0000355645), CCGGCACGTTAACAGTGGGTGCCAACTCGAGTTGGCACCCACTGTTAACGTGTTTTTG (TRCN0000355646) and CCGGTTCTGGCCTGCCTTCGTTAACCTCGAGGTTAA CGAAGGCAGGCCAGAATTTTTG (TRCN0000355647). HEK293FT cells were co-transfected with shRNA plasmid together with the packaging plasmids pLP1, pLP2 and pVSVG. After 48 h, the virus-containing supernatant was harvested and then infected target A549 cells. Stable cells were selected with 8 μg/ml puromycin 48 h after infection. The stable cells from a mixed population were used for c-Jun shRNA screening. Clone TRCN0000355646 and the pLKO.1-puro non-target shRNA control (Sigma) were used in this study (Supplementary Figure S3).

#### RNA-Seq sample preparation, sequencing and data analysis

A549 cells were stimulated in the absence or presence of TNFα (30 ng/ml, 0.5 h, 1 h, 3 h or 6 h) and subjected to total RNA extraction with TRI Reagent (Sigma). Five micrograms of the isolated RNA was treated by RNase-free DNase I and further depleted of ribosomal RNA by use of Ribo-Zero rRNA removal kit (Epicentre). After thermal fragmentation with cation, RNA samples were subjected to cDNA synthesis using reverse transcriptase (Super-Script II) and random primers. The cDNA was further converted into double-stranded DNA using NEBNext mRNA second strand synthesis module (NEB), the resulting dsDNA was used for bar-coded library preparation using NEBNext DNA Sample Prep Master Mix Set 1 (NEB). Every three indexed libraries were pooled together and multiplexed pair-read runs were carried out with 75 cycles per run on an Illumina HiSeq 2000 instrument (Illumina, Inc.) in the UT Southwestern Genomics Core.

We used well-established software package to estimate transcript levels in our RNA-Seq data sets. Briefly, Bowtie and Tophat (34) were used to align reads to the human genome and discover transcript splice sites. These alignments were assembled into transcripts by Cufflinks (34). Cuffdiff, a component of the Cufflinks suite, was used to compare the aligned reads from multiple conditions to find differentially expressed genes.

## RESULTS

Our goal was to propose a probabilistic method for highly accurate reconstruction of the NF-κB/RelA cistrome and identifying *cis*-regulatory modules controlling its action. We were also interested in elucidating the local chromatin context associated with functional NF-κB/RelA binding and in inferring principles controlling the expression of the NF-κB/RelA regulatory network. To accomplish this, we constructed a computational pipeline, visualized in Figure 1. The input data were obtained using a highly efficient ‘two-step’ cross-linking chromatin immunoprecipitation (XChIP) method (29). Using our in-house Instant Sequencing and MACS software, we processed ChIP-Seq data and identified most likely locations of NF-κB/RelA TFBSs. The enrichment of sequence motifs in NF-κB/RelA TFBSs was determined using an exhaustive *de novo* motif search (‘Materials and Methods’ section). Thus, computationally found putative TFBSs of TFs other than NF-κB/RelA were validated by comparing with the recent ENCODE ChIP-Seq data sets for the corresponding TFs. NF-κB/RelA-regulated gene assignments were based on both our probabilistic comprehensive approach and a more traditional approach, including only genes with NF-κB/RelA TFBSs in their promoters (Supplementary webpage, files B and C). Functions and expression patterns of NF-κB/RelA-putatively regulated genes are discussed. We show that our probabilistic approach to inferring target genes for TFBSs leads to more convincing set of NF-κB/RelA-regulated genes, as judged both based on analysis of their GO functions and expression patterns upon TNF stimulation.

### Generation of NF-κB/RelA cistrome data by XChIP-Seq

The experiment used human type II alveolar epithelial (A549) cells stimulated with TNFα (30 ng/ml for 30 min), conditions where NF-κB/RelA is efficiently activated (11,28). Because earlier studies have shown that NF-κB is in hyperdynamic exchange with its target high-affinity binding sites in chromatin (35), we have developed and optimized a highly efficient sequential protein–protein followed by protein–DNA cross-link method. XChIP produces highly efficient and quantitative cross-linking of NF-κB/RelA and related transcription factors to chromatin, without producing cross-links with non-NF-κB/RelA-binding segments of DNA (14,28).

XChIP sequencing data were pre-processed and filtered (‘Materials and Methods’ section); the reads were then mapped to the human genome (GRCh37/hg19). Within these data, we identified 77% and 81% unique sequence reads with zero mismatches (U0) in control and TNF stimulated samples, respectively (Supplementary Table S1). Overall, ∼90% of the reads could be mapped with zero mismatches in both samples (Supplementary Table S1). The number of poly-A reads (4–6%) is consistent with the average genome frequency (4%). We have also examined quality score distribution within reads, which showed overall high-quality and expected trends. These results indicate high quality of our data set. Using MACS software (3), we identified 20 733 total peaks in these data, which are representing putative NF-κB/RelA-binding regions.

To independently assess the quality of the peaks, we rank-ordered the top-scoring 20% peaks based on the MACS quality scores, and we removed previously known NF-κB targets. Twenty-one novel regions were selected for qualitative validation, 11 from the top of the rank-ordered peak list and 10 from the bottom of the peak list. Q-gPCR of XChIP-prepared genomic DNA was performed on an independent experiment from control and TNF-stimulated A549 cells. We were able to unambiguously confirm enrichment of NF-κB/RelA binding for all 11 of the top of the novel rank-ordered list (Figure 2A). For example, a 60-fold enrichment of NF-κB/RelA binding was observed in the TNF-treated chromatin relative to that of unstimulated cells for RelA_Peak_154. The level of NF-κB/RelA enrichment varied, with the lowest being a 4-fold enrichment observed for RelA_Peak_1510 and RelA_Peak_3929 (Figure 2A). The fact that all 11 RelA TFBSs tested were also validated indicates that false-positive ratio among our high-quality peaks is likely <6%. The frequency of validation was reduced among the lowest quality peaks, where 5 of the 10 genes were validated through showing enrichment by XChIP-Q-gPCR (Figure 2B). These results underscore the overall quality of the data and show good correlation of NF-κB/RelA binding with the peak quality scores. Because of the very low false-positive rate of the top 20% of putative NF-κB/RelA TFBSs, we focus the subsequent study on the 4195 highest-quality binding regions (Supplementary webpage, file A).

### Assignment of NF-κB/RelA-regulated genes

We next analyzed the location of the NF-κB/RelA peak summits relative to the closest TSS of known genes, using conventional method of assignment of a target gene to a given NF-κB/RelA TFBSs (‘Materials and Methods’ section). We observed that there is clear enrichment of peaks within 2 kb of the TSSs, in a manner that is slightly different for upstream and downstream regions (Figure 2C). A histogram depicting their relationship informed the design of our probabilistic prediction of NF-κB/RelA target genes. Namely, we have divided the possible TFBS locations relative to TSS into 16 possible categories, based on both distance from TSS, upstream or downstream of TSS location or location in intron or exon (Table 1). For each category, we computed the enrichment of NF-κB/RelA peaks in such a configuration versus randomly simulated positions of NF-κB/RelA peaks in the whole genome (Table 1). The regulation odds thus learned from the data presented a different picture than the histogram derived with popular, but naïve method of assigning gene with TSS nearest to a TFBS as being regulated. Our calculations revealed a strong preference of regulatory NF-κB/RelA TFBSs to be in close proximity (<2 kb) of the regulated gene TSS upstream (enrichment value of 6.73). The second most favored position for regulatory NF-κB/RelA motifs was in introns and in distance smaller than 2 kb from the TSS (enrichment value of 4.37) (Table 1). The third most likely location is within 2-kb downstream. The least favored positions for NF-κB/RelA TFBSs were further than 1 Mb from a closest TSS, irrespective of the intron, exon or intragenic location.

To identify a gene most likely to be regulated by a given NF-κB/RelA TFBS, we used different likelihoods for NF-κB/RelA TFBSs to regulate nearby genes depending on their relative orientation and chromatin context. Then, for each gene, we combined contribution of all NF-κB/RelA TFBSs that most likely regulate that gene to achieve final scoring.

The NF-κB/RelA-regulated gene list produced by assignment to the nearest gene within ±2 kb of the NF-κB/RelA TFBS (traditional method) has only a 40% overlap with the list produced by our probabilistic assignment (Supplementary Tables S4 and S5 and Supplementary webpage, files B and C). Additionally, comparison of GO enrichments showed that the probabilistic scoring yielded a much higher informativity score (Supplementary Figure S2), indicating that false-positive assignments have been reduced by our method. Surprisingly, the second highest scoring gene on our more accurate list of NF-κB/RelA targets was TANK, a gene previously unknown to be NF-κB/RelA regulated. Later in the text we describe how we comprehensively validated that TANK is indeed a genuine NF-κB/RelA target gene, underscoring usefulness of our computational method.

### Identification and validation of TANK as a *bone fide* NF-κB/RelA target

Our GO molecular functions enrichment analysis and top-scoring molecular pathways identified by the IPA knowledge base indicated that at least 15 genes in the NF-κB pathway itself were identified as NF-κB/RelA targets. A characteristic of the NF-κB/RelA pathway is that it is under negative autoregulatory control, where its activation induces negative feedback regulators, including the IκB family of inhibitors (BCL3, IκB-α, -β, -γ, -ε) and NF-κB-1 and 2. However, our analysis identified additional sites of autoregulation, including the TRAF family member associated NF-κB activator (TANK), and IκB kinase β (IKKβ; Figure 6A).

TANK is a cytoplasmic scaffold that recruits downstream IKKs to activated TNF receptors. To validate its potential regulation by NF-κB/RelA, we examined the NF-κB/RelA ChIP peaks in the vicinity of the TANK gene; three high-scoring TFBSs were identified in the region 5′ to the TSS (Figure 6B). Also of note, is the co-occurrence of AP1 and break point motif in this promoter (Figure 6B). To experimentally validate the inducible binding of RelA and AP1, XChIP-Q-gPCR experiments were performed in control and TNF-stimulated A549 cells. Here, we observed a 7-fold enrichment of RelA and a 2-fold enrichment of AP1 on the TANK promoter in TNF-stimulated chromatin relative to unstimulated cells (Figure 6C). We also examined the temporal profile of TANK mRNA in response to TNF stimulation. We observed that TANK mRNA was 3.5-fold inducible 3 h after TNF stimulation in A549 cells (Figure 6D).

To demonstrate that the induction of TANK was dependent on NF-κB/RelA, we measured TANK mRNA changes in TNF-stimulated HeLa^tTA/FLAG-IκBα Mut^ cells, where NF-κB/RelA activity can be controlled exogenously by the tetracycline transactivator (9,11). In the presence of the tetracycline analog, doxycyline, HeLa^tTA/FLAG-IκBα Mut^ cells are functionally wild-type; however, in its absence, HeLa^tTA/FLAG-IκBα Mut^ cells express the non-degradable IκBα site mutation (FLAG-IκBα Mut) at levels that completely inhibit NF-κB translocation and target gene expression (9,11). We found that NF-κB blockade significantly inhibits TNF-induced TANK mRNA expression compared with the induction of TANK in the presence of doxycyline (Figure 6E). These results demonstrate that activation of NF-κB/RelA pathway is required for TANK mRNA expression; therefore, TANK is a *bone fide* NF-κB/RelA target.

### NF-κB/RelA Gene Ontology (GO) annotations

To identify biological processes under control of NF-κB/RelA, we identified 787 genes with TSSs within 2 kb distance of the top 20% NF-κB/RelA peaks and subjected this list to two different GO analyses. The first analysis involves identification of enriched or depleted GO terms in biological process, cellular component and molecular function categories using the Informativity Calculator (http://informativity.utmb.edu). From this analysis, 100 significant GO biological processes were identified that included the well-established effects of NF-κB/RelA: regulating programmed cell death/apoptosis, nucleocytoplasmic transport, adaptive immune response, cascade and platelet-derived growth factor pathway, response to DNA damage, transcription upregulation and the NF-κB-IκB signaling cascade, a feature also identified in the cellular component analysis (Supplementary Table S3 and Supplementary file D on the webpage). The molecular functions were enriched in small molecule GTPase activity, including the Rho and Rac pathways controlling cell motility. We noted that NF-κB pathway, DNA repair and cell cycle regulation were biological processes that tended to be enriched more in genes whose NF-κB/RelA-binding sites were located within ±2 kb of the TSS. Interestingly, on ultra short distances—up to 100 nt—the most significantly enriched GO function is base excision DNA repair. NF-κB pathway genes have slight preference for having a RelA TFBS within ±250 nt of the gene’s TSS. Moreover, the analysis also yielded significantly enriched GO function previously not described as regulated by NF-κB, such as regulation of cell motility. We have also noted that genes with putative NF-κB/RelA-binding site farther away than ±2 kb from the gene start exhibited highly non-random GO functional annotations, supporting our approach to include such genes in RelA regulatory network.

**Figure 3.**
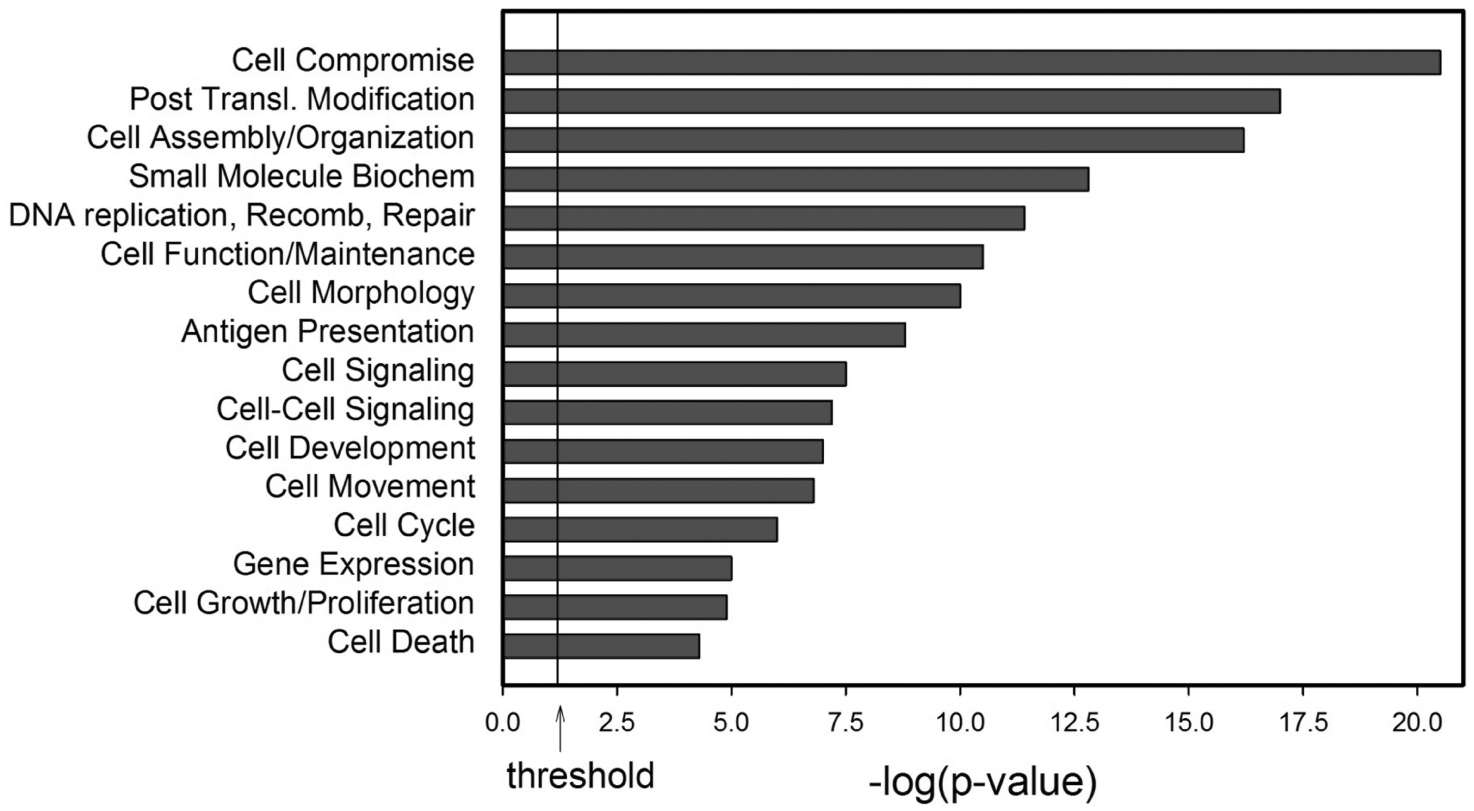
Genome Ontology. Shown are the molecular functions of 787 genes within 2 kb of the top 20% MACS peaks by Ingenuity Pathways Analysis (www.ingenuity.com). The *y*-axis is the molecular function; *x*-axis is −log(*P*-value) for the enrichment of that activity over the human genome.

A second approach to GO analysis involved the identification of biological processes using the Ingenuity Pathways Knowledge base, an expert-curated summary of gene and protein interactions from the peer-reviewed literature (Figure 3). Cell death, cell-to-cell signaling and interaction and antigen presentation are significantly enriched and known biological processes controlled by NF-κB, including the anti-apoptotic genes A20/TNFAIP3, Bcl-Xl, cytokine and chemokine networks and transporters of antigen peptides and major histocompatibility complex molecules. In addition, cell morphology and cell migration molecular functions were identified, containing the Rac/Rho GTPases represented in the GO molecular function analysis (compare Supplementary Table S3), providing more support for NF-κB/RelA involvement in the regulation of cell motility.

**Table 2.** *De novo* motif enrichment

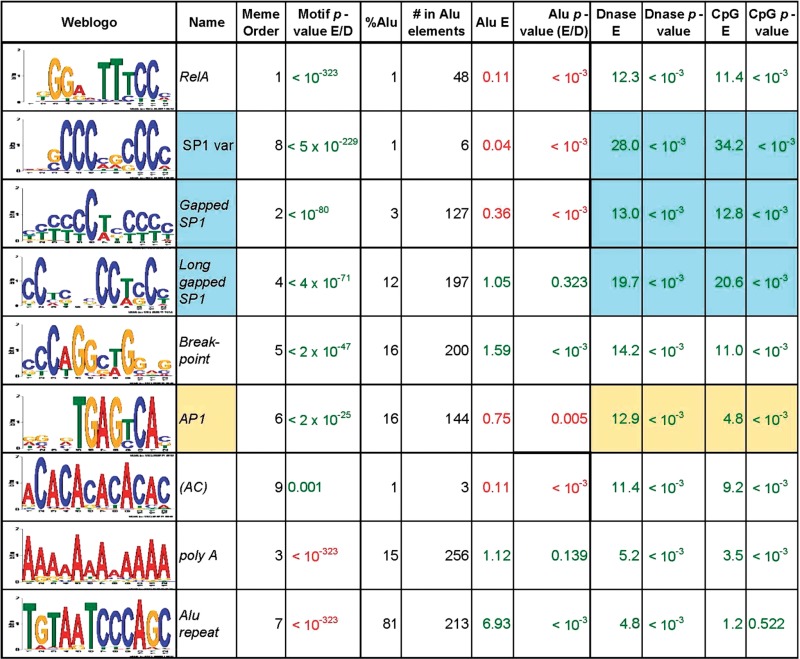

Shown are the nine top motifs from *de novo* analysis of 500-nt sequences from 4195 of NF-κB/RelA ChIP-Seq peaks (representing the highest 20% significance scores). Column 1, the Weblogo for the 11 nt *de novo* motif search; 2, motif name; 3, motif rank according to MEME; 4, the corrected empirical *P*-value computed by us (green indicates enrichment, red indicates depletion); 5 and 6, percentage and number of motif occurrences within Alu repeats; 7 Alu repeat enrichment; 8, *P*-value for Alu enrichment (green) or depletion (red); 9 and 10, enrichment of DNase hypersensitive sites (Genome Browser OpenChromSynth_K562_DNase track) and its *P*-value; 11 and 12, enrichment of CpG islands (Genome Browser CpG Islands track) and its *P*-value.

### *Cis*-element enrichment in NF-κB/RelA-binding regions

We next conducted an exhaustive *de novo* motif search. Motif search was performed in a broad vicinity (±250 b) of the RelA ChIP-Seq peaks summits using MEME v. 4.4. We did not rely on the default MEME E-value to estimate statistical significance of the motifs, as it can substantially overestimate the significance of low-complexity sequences, even reporting as significantly enriched motifs that are actually depleted (Table 2). Instead, the statistical significance of enrichment of each motif was calculated using its actual genome frequency (‘Materials and Methods’ section). This *de novo* approach allows for discovery of novel motifs, and our empirical probability estimation allows for accurate detection of truly overrepresented motifs.

**Figure 4.**
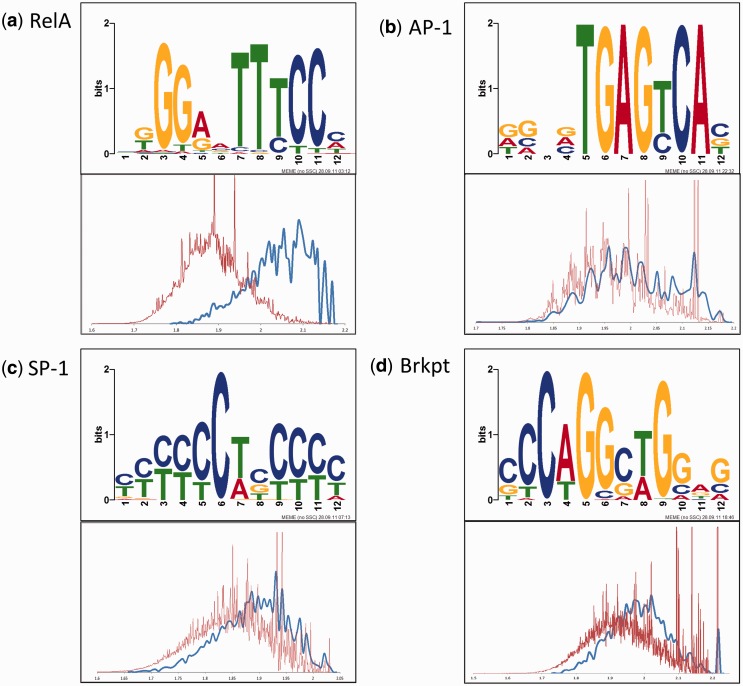
Motif enrichment. Shown are the weblogos for four primary *cis*-regulatory motifs of 12-nt length identified in the MACS peaks (**a–d**, motifs of NF-κB/RelA, AP1, gapped SP1, break point and their validation histogram, respectively). The blue line depicts the distribution of the likelihood scores (based on log-transformed position weight matrix) for a given motif to occur among the top 20% of the RelA Chip-Seq peaks ChIP peaks, and the red line depicts the same distribution for the whole genome. For each motif, the validation histogram is plotted, which clearly shows that the given motif is genuinely enriched in RelA peaks as compared with the whole human genome. The motifs denoted as depleted in Table 2 do not pass this test.

First, we searched for 12-nt motifs among the 500-nt sequences centered on the NF-κB/RelA peak summit in the top 20% of the rank-ordered MACS peaks. Strikingly, MEME consistently found statistically significant occurrence of the canonical NF-κB/RelA motif (Figure 4A) for all 4195 analyzed peaks. Using *P*-value corrected based on actual genomic frequency of each motif (Table 2), high-confidence motifs were identified in 3202 peaks. Because we have only analyzed in detail ∼20% of the peaks, this result indicates there are up to ∼16 000 high-quality NF-κB/RelA-binding sites among regions revealed as differentially bound in our XChIP-Seq data set. The most significantly enriched sequence motif is 5′-KGGRNTTTCCM-3′ (Figure 4A, top panel), a sequence that corresponds closely with the consensus NF-κB/RelA sequence of 5′-GGGRNTTTCC-3′ identified by an *in vitro* selection technique from a degenerate oligonucleotide library (19). This motif is truly enriched in the RelA ChIP-Seq peaks (as opposed to having significant theoretical MEME E-value), as confirmed by comparison of likelihoods of this motif occurrences in these peaks (blue histogram, Figure 4A) and in the human genome (red histogram, Figure 4A).

**Figure 5.**
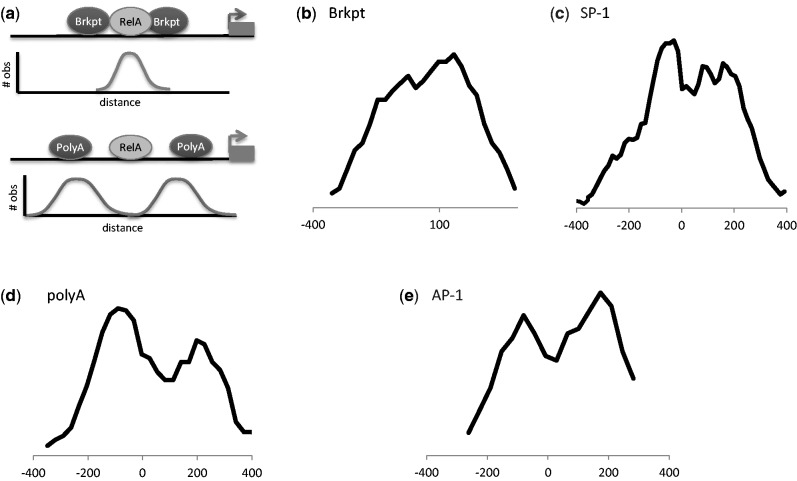
Intra-motif distance histograms. (**A**) Schematic diagram. The highest significant NF-κB/RelA summit is centered at 0 in the analysis. (**B**–**E**) Histogram of distances between RelA motifs and breakpoint, SP1, poly A and AP1 motifs, respectively.

Similarly, the actual enrichment in the RelA ChIP-Seq peaks as compared with the human genome was observed for 5′-TGAGYCA-3′ (matching a consensus AP1-binding site, Figure 4B) and 5′-YYYCTRYYY-3′ {matching a gapped SP1 consensus binding site [Figure 4C, (36)]}. Our *de novo* approach also identified 5′-CCAGCCTGG-3′, a motif associated with DNA conformational changes known as a break point motif [Figure 4D, (37)]. Although the enrichment of these sequences was not as high as observed for the NF-κB/RelA consensus site, all were significantly overrepresented in the XChIP-Seq data set (Table 2).

Nine sites were found to be significantly enriched in the top 20% of the NF-κB/RelA MACS peaks by MEME (Table 2). Interestingly, three of the motifs reported by MEME as significantly enriched based on theoretical calculations were in reality either borderline enriched [(AC)n] or strongly depleted (poly A and Alu repeat motifs), as shown by our empirical *P*-value correction (Table 2, ‘Materials and Methods’ section). The depletion of the poly A motif may be related to the fact that poly A sequences are enriched at the 3′-end of genes, where they play important roles in signaling pre-mRNA processing and export. Alu repeats are retrotransposon sequences that are also enriched in poly A repeats and are also naturally depleted in the close proximity of the NF-κB/RelA-binding sites. We will discuss interplay between Alu repeat and NF-κB/RelA binding in more detail later in the text.

We further systematically searched for motifs between 7 and 25 nt of length in the top 20% of NF-κB/RelA ChIP-Seq peaks. All these searches returned as a top result motifs consistent with the canonical NF-κB/RelA-binding site, which proved to be well-defined 11-nt island of informative positions with a 9-nt core (Figure 4A, and see also Supplementary Figure S1A). Even though the AP1 and gapped SP1 motifs were recognizably similar in searches for motifs of different lengths, they seemed slightly differently in longer motif searches. The AP1 motif shows strong nucleotide preferences at six of seven core positions (Table 2 and Supplementary Figure S1B). The novel DNA break point motif extends to 25-nt motif with equally strong nucleotide preferences, suggesting its characteristic sequence is at least 25-nt long. We also identified three variants of the SP1 motif; the most significant was a gapped variant flanking triple Cs. This analysis reveals the flexible nature of the SP1 sequences, and it becomes obvious that the gaps between C residues can vary substantially (Table 2 and Supplementary Figure S1).

### D*e novo* motif validation and further characterization of RelA TFBSs by using ENCODE data

Our analysis revealed that NF-κB/RelA ChIP-Seq peaks were enriched in several variants of SP1 sequence motifs and AP1 sequence motif (Table 2). To verify whether these sequence motifs correspond to TFBSs *in* vivo, we turned to the recent ENCODE data on experimentally derived binding sites for 119 human TFs. Careful enrichment analysis, taking into account TFs tendency to bind in non-random locations in the human genome (‘Materials and Methods’ section), confirmed that the corresponding TFs binding indeed correlates with the enrichment of sequence recognition motifs we found (Table 3 and file E on the Supplementary webpage). The predicted AP1 TFBSs were significantly enriched in genomic regions experimentally identified by ENCODE to bind AP1 subunits: AP-2, c-Jun and JunD (Table 3). Similarly, predicted AP1 motif-enriched chromatin was enriched in glucocorticoid receptor (GR)-binding sites, an observation validated by separate ChIP-Seq study (27).

**Table 3.** Enrichment of ENCODE transcription factor measured binding

	Enrichments of ENCODE transcription factor binding									
	AP-2 alpha	AP-2 gamma	c-Jun	JunD	SP1	STAT1	CTCF	BAF155	GR	BCL3
SP1 var	106	96	34	35	56	65	11	155	84	61
Gapped SP1	68	64	42	39	38	60	7	91	74	41
Long gapped SP1	77	71	38	36	46	59	10	122	79	45
AP1	94	90	97	100	43	82	5	154	125	56

Selected ENCODE transcription factors and four *de novo* motifs (three dominant variants of SP1 motif and AP1 motif) are shown, full list is available on Supplementary webpage. Note that binding of AP1 subunits, as measured by ENCODE consortium, is especially enriched in location where we detect AP1 sequence motifs. Moreover, RelA ChIP-Seq peaks exhibit different enrichments of binding of many TFs examined by ENCODE, depending on the presence of SP1 motifs or AP1 motif. For some transcription factors, SP1 motifs with middle ‘T’ (gapped SP1 and long gapped SP1) exhibit different co-occurrence preferences than SP1 var motif.

### Motif occurrence inside Alu repeats

Based on a data set several orders of magnitude smaller than ours, it was recently suggested that NF-κB/RelA binding preferentially occurs in Alu repeats and that a novel Alu-κB motif is actually the most common NF-κB/RelA-binding site (38). Therefore, we analyzed the frequency of Alu repeat sequences among NF-κB/RelA ChIP peaks, as well as occurrence of Alu-κB motifs within them. First, we found that the consensus Alu-κB site was extremely underrepresented in our data. Moreover, using Repeat Masker database, we found that the overall frequency of repetitive elements in ±250 nt vicinity of summits of NF-κB/RelA peaks is half of the genome average, and that the frequency of Alu repeats is less than one-third of the average.

We have also performed a more direct analysis and computed how often NF-κB/RelA motif occurs within an Alu repeat (Table 2). This analysis of frequency of motif occurrence within repetitive sequences shows that the NF-κB/RelA motif occurs rarely (1%) within an Alu repeat. This finding contrasts with the occurrences of the long gapped SP1 variant, AP1 and break point motifs, motifs that occur within Alu repeat with frequency between 12 and 16% (Table 2). Finally, we use our simulations method (‘Materials and Methods’ section), to assess Alu repeat enrichment or depletion in RelA peaks, which again indicated significant depletion of RelA peaks in Alu repeats.

Additionally, one of the motifs found by MEME in our *de novo* search is part of an Alu repeat (‘Alu repeat’, Table 2). This motif, after re-computing true significance, turn out to be significantly depleted among NF-κB/RelA ChIP peaks. Moreover, the Alu repeat motif is the only motif on our list that statistically significantly ‘avoids’ co-occurring with NF-κB/RelA (Table 4) providing even more evidence against Alu repeat enrichment in NF-κB/RelA ChIP peaks. All these results make it unlikely that the true frequency of NF-κB/RelA binding within Alu repeat is 11%, as claimed in the recent article (38). The result reported previously (38) maybe an artifact of the cloning technique used or of the very small sample size.

**Table 4.** Motif correlation analysis

Motif #	RelA	Long Gapped SP1	PolyA	Alu	BrkPoint	SP1-var	AP1	Gapped SP1
RelA		0.21	1.89	6.60D	0.17	0.52	2.48	0.00
Long Gapped SP1			5.87D	29.90E	50.58E	27.50E	30.42D	32.76E
PolyA				39.83E	0.24	53.27D	62.85D	11.40D
Alu					64.08E	5.95D	1.00	40.29E
BrkPoint						29.91E	9.06D	74.08E
SP1-var							45.82D	16.80E
AP1								16.27E
Gapped SP1								

For each motif in Table 2, *χ*^2^ correlations with other motifs in the top 20% RelA ChIP peaks are shown. D, depleted; E, enriched. Note that AP1 is depleted in the presence of any other motif found.

Finally, the novel DNA break point motif occurs often (16%) within Alu repeat and in 31% of cases within a repeat region. Repetitive regions are known to be especially fragile; therefore, presence of DNA break point motif in such an environment is not surprising.

### Motif relationships

We next examined spacing and correlations of the sequence motifs found to elucidate their relationship with NF-κB/RelA TFBSs. Previous functional assays of NF-κB-dependent genes have suggested that the spacing of other inducible enhancer motifs control the kinetic profile of target gene activation. To determine whether the locations of NF-κB/RelA binding were randomly distributed or spatially constrained relative to the other enriched motifs, we analyzed the distribution of their locations with respect to the NF-κB/RelA motif in the top 20% NF-κB/RelA ChIP peaks. We found that the break point motif was closely overlapping with the NF-κB/RelA motif (schematically diagrammed in Figure 5A, and distribution shown in Figure 5B). The gapped SP1, the poly A and the AP1-binding site were also bimodally distributed, but at differing characteristic distances, located −100 nt and +100 nt relative to that of NF-κB/RelA (Figure 5C–E). We conclude that the AP1, gapped SP1 and break point motifs are spatially constrained relative to the location of NF-κB/RelA motifs, which suggest that they physically interact. We have also used the recent ENCODE data to confirm that AP1 and SP1 subunits are indeed binding in the location where we identified their binding motifs in the NF-κB/RelA peaks (Table 3 and Supplementary webpage file E).

An analysis of motif co-occurrence is shown in Table 4. From this *χ*^2^ analysis, we see that the only statistically significant preference of the NF-κB/RelA motif is to avoid the Alu repeat. The long gapped SP1 motif significantly co-occurs with Alu, break point and SP1-variant motif, a finding that underscores the flexible binding preferences of this transcription factor. The co-occurrence of long gapped Sp1 with the Alu repeat motif is also confirmed by our earlier analysis of motif placement with respect to repetitive elements (Table 2). Interestingly, the SP1-variant motif, the most significantly overrepresented SP1 family motif (Table 2), is depleted with AP1 and the poly A motifs, the latter representing a motif underrepresented in all NF-κB/Rel A peaks, except in those containing Alu repeats (Table 4). By contrast, the occurrence of AP1 motif in the NF-κB/RelA enriched sequences is depleted with respect to SP1 motifs (Table 4). These data suggest that RelA chromatin segregates into two separable domains, differentiated by the presence of either SP1 and or AP1 motifs.

### Different NF-κB/RelA sub-pathways induced by RelA interaction with AP1 or SP1

Our *de novo* motif search established motifs similar to canonical AP1 site and several variants of SP1 motifs as main secondary motifs found in NF-κB/RelA peaks (Table 2). Moreover, using the ENCODE data set, we have found that that our estimates of sequence motif enrichment highly correlate with actual binding of the corresponding TFs within the NF-κB/RelA peaks (Table 3 and Supplementary webpage, file E). We have also observed that co-occurrence of NF-κB/RelA with either of the AP1 or SP1 motifs correlates with different chromatin context (preferences for open chromatin or intron location), different GO functions and different expression pattern of regulated genes (Figure 7 and Figure 8).

### RelA-AP1 and RelA-SP1 regulated genes have distinct expression profiles

To obtain preliminary indication of whether the co-occurrence of Rel with AP1 or SP1 had functional consequences, we conducted a time series measurement of global RNA expression profiles in TNF-stimulated cells using RNA-Seq. The time series profiles of genes controlled by RelA-AP1 combinations were combined and compared with those genes controlled by RelA-SP1 combination. Here, we noted that the RelA-SP1-regulated genes were maximally activated by 0.5 h, and fell thereafter (Figure 7). By contrast the RelA-AP1 genes peaked at 1 h of stimulation and declined thereafter. These data indicate that the co-occurrence of AP1 or SP1 is related to differential timing of NF-κB/RelA-regulated subnetwork expression.

**Figure 6.**
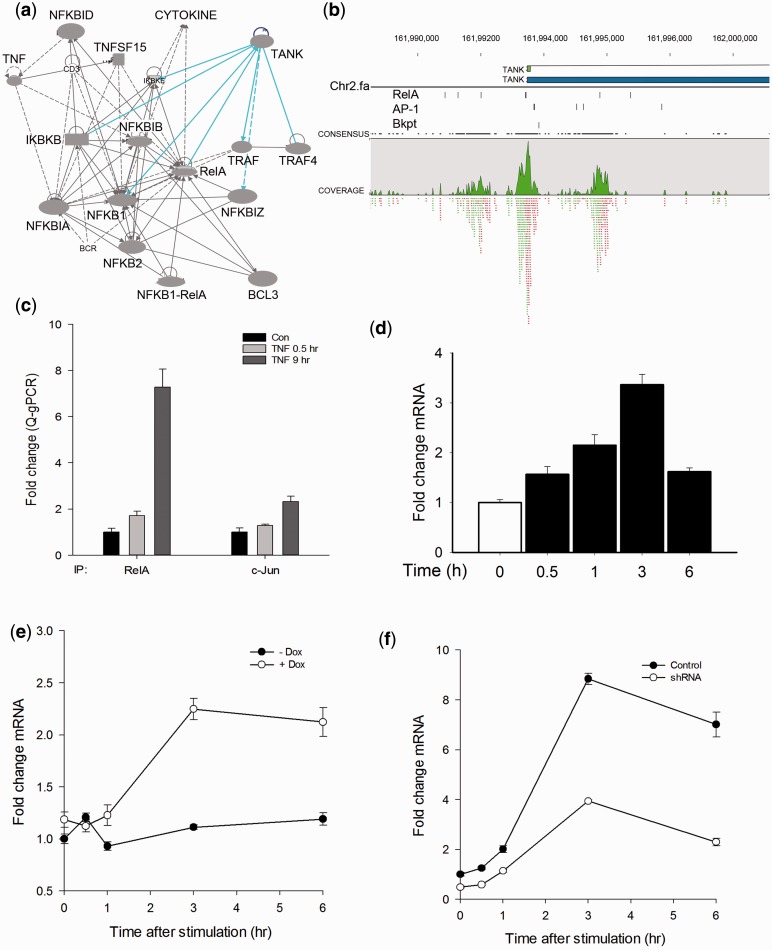
Validation of TANK as an NF-κB/RelA target. (**A**) Ingenuity pathway network of NF-κB family members identified in the 20% of significant peaks. Each node is a gene; edges indicate protein–protein interactions (solid lines) and activation (directed arrow). Abbreviations are BCL3, B cell CLL/lymphoma 3; BCR, B-cell receptor; NFKID, IκB-Δ; NFKBIZ, IκB-ζ; TNFSF15, TNF secreted ligand 15 kDa; NFKBIA, IκBα, IKBKB, Inhibitor of IκB kinase β; IKBKE, IκBε; TANK, TRAF family member associated NF-κB activator; TRAF, TNF-associated factor. (**B**) Top panel, location of NF-κB/RelA peaks on the TANK gene. Shown are TANK gene 5′-UTR, TSS and TANK exons as small green boxes. Bottom panel, NF-κB/RelA peaks (green). (**C**) Validation of NF-κB/RelA and AP1 binding. XChIP was performed on control or TNF-stimulated A549 cells. Chromatin was immunoprecipitated using RelA or AP1 Abs as indicated. Shown is fold enrichment of TANK promoter sequences by Q-gPCR. (**D**) Induction of TANK mRNA expression. A549 cells were stimulated with TNF, and TANK mRNA quantified by Q-RT-PCR. Shown is fold change in normalized TANK mRNA as a function of time. (**E**) Validation of NF-κB dependence on TANK expression. HeLa^tTA/FLAG-IκBα Mut^ cells were plated in parallel in the absence (−Dox) or presence (+Dox) of doxycyline (Dox, 2 μg/ml) and stimulated with TNF. TANK mRNA abundance was determined by Q-RT-PCR. (**F**) AP1 knockdown effect on TANK expression. A549 cells were transfected by lentiviral control shRNA (pLOK.1) or c-Jun shRNA and the pooled puromycin-resistant cells were further treated by TNF and analyzed TANK expression by Q-RT-PCR. Shown is normalized fold change mRNA expression.

Our motif enrichment and RNA-Seq analyses suggests that the co-association of AP1 affects the regulation of a subset of NF-κB/RelA-regulated genes. To address the functional involvement of AP1 in TNF-induced TANK activation, we performed a knockdown of AP1 by expressing c-Jun shRNA in A549 cells. Highly efficient knockdown of c-Jun was determined by selection of candidate shRNAs (Supplementary Figure S3). Here, c-Jun depletion resulted in 40–70% decrease in TNF-induced TANK expression levels measured by Q-RT-PCR (Figure 6F). Interpreted together with the finding that c-Jun enrichment was found on TANK promoter after TNF stimulation (Figure 6C), this result indicates that NF-κB and AP1 cooperate in the induction of TANK expression. Together, these findings extend the spectrum of feedback regulators controlling the NF-κB pathway to include the TANK scaffolding protein whose expression is modulated by a functional AP1 interaction.

**Figure 7.**
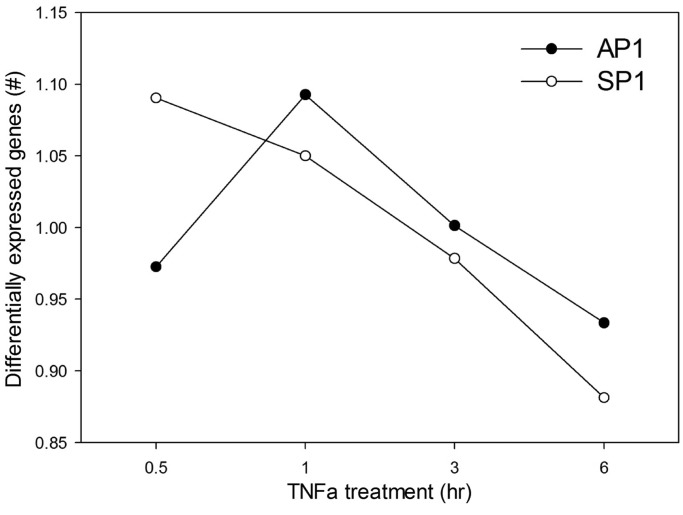
Expression patterns of genes controlled with RelA-AP1 or RelA-SP1 motifs. The total number of differentially expressed genes relative to unstimulated cells was derived from RNA-Seq of a time course experiment. The percentage of differentially expressed genes with AP1 or SP1 motif was calculated and normalized by the average percentage of genes with the given motif across all the time points.

## DISCUSSION

**Figure 8.**
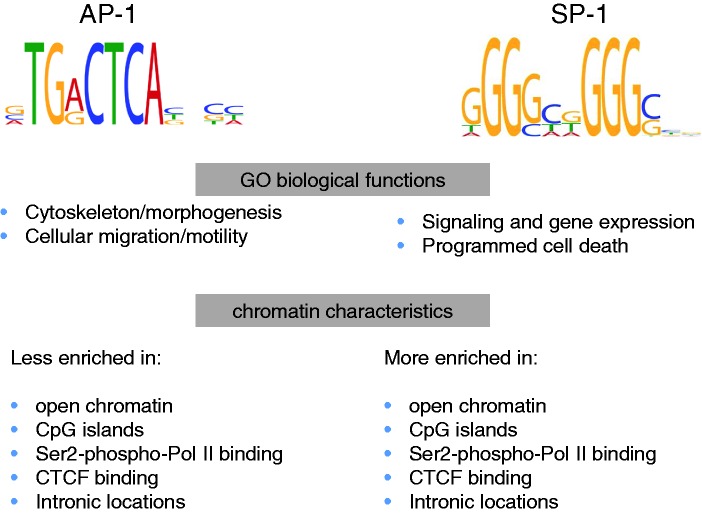
Segregation of NF-κB/RelA network into distinct functional chromatin environments. Statistical enrichment studies show that NF-κB/RelA-AP1- and NF-κB/RelA-SP1-enriched motifs segregate into distinct populations. These two populations were analyzed by mapping to candidate genes and analyzing biological function and comparing enrichment with chromatin modifications available within ENCODE. For each population, the distinct GO biological function and chromatin characteristics are shown.

One of the major challenges in computational systems biology is an accurate reconstruction of regulatory networks. Even though significant progress has been made in experimental high-throughput detection of the binding sites using ChIP-Seq, less improvement has been made in integrating the data to achieve accurate information of putative binding sites for a given TSS. Here, we propose an approach to reconstruct the NF-κB/RelA cistrome, identify *cis*-regulatory modules controlling its action, elucidate information about the local chromatin context in which it operates and identify properties of the NF-κB/RelA regulatory network. Our approach is novel in that we propose a method to learn preferred relative localizations of the functional binding sites to corresponding TSSs of the regulated genes with respect to more accurately assign a TFBS to a gene it most likely regulates. Moreover, we integrate the contributions of all TFBSs most likely regulating a given gene to achieve the final regulation score. This way, a gene target regulated by several not strong TBFSs—such as the novel NF-κB/RelA regulated gene TANK—can also be reliably found. Although our analysis indicates that even higher accuracy of calling functional binding sites could be achieved by using various chromatin features (such as chromatin accessibility, Supplementary webpage), in the present article, we restrict ourselves to discuss how to reconstruct high-accuracy regulatory network using only ChIP-Seq data against single antibody, to make our work more broadly useful. As a proof of concept, we present how our method works for uncovering the regulatory network of NF-κB/RelA transcription factor, a TF that serves as a master regulator of inflammation, anti-apoptosis and regulatory signaling ubiquitously in host cells. We combine our NF-κB/RelA ChIP-Seq data with the results of 119 ChIP-Seq experiments performed by the ENCODE consortium, to confirm our *in silico* findings that NF-κB/RelA interacts with distinct chromatin domains. RelA-SP1 domains occur in DNase I-accessible intronic binding sites associated with phospho-Ser2 Pol II-enriched chromatin, whereas RelA-AP1 domains occur in a more closed chromatin environments (schematically illustrated in Figure 8).

Previous work has found functional motifs in NF-κB/RelA-dependent promoters of variable distance from the TSS (11), with some binding sites located within downstream introns (such as the Igκ gene), or up to 10-kb upstream in the TNF promoter. Our analysis of the occurrence of NF-κB/RelA binding relative to known structural elements of genes indicates that NF-κB/RelA can be widely distributed in introns, exons and extra-genetic locations, although the chances of these binding sites to be functional highly depend on their localization (Table 1). For example, in our estimation the RelA binding motif located <2-kb upstream from the TSS is almost 40-times more likely to regulate the given genes than would be the same motif located in intron. Analogously, the motif located 2-kb upstream of the TSS is ∼20 more times more likely to regulate a given gene compared with the same motif located >1 Mb upstream of that TSS (Table 1). Our final scoring of the genes according to their chances of being RelA regulated includes the contributions of all NF-κB/RelA motifs putatively regulated by a given gene, contrary to common practice of including only close motifs, present even in the recent probabilistic approach (6).

Moreover, unlike in the mentioned computational model, high-quality TFBS are identified using leading peak-calling software MACS, not based on less sophisticated method of TFBS scoring based on calculating overlap in read pattern between a given TFBS and averaged values for all TFBSs of the considered TF.

The value of our method is well illustrated by our prediction that TANK—the second highest scoring NF-κB/RelA-regulated genes according to our calculations—is NF-κB regulated; TANK has not previously been identified as being NF-κB/RelA dependent. In this article, we confirmed this prediction experimentally. Moreover, the set of NF-κB/RelA-regulated genes found by our method highly correlates with differentially expressed genes found in RNA-Seq data after TNF treatment (*P*-value = 10^−^^55^, hypergeometric test) and has high biological informativity (*P*-value = 0.004, computed using Informativity tools). Both of these comparisons are favorable for our method of uncovering NF-κB/RelA target genes, as compared with traditional method of only considering TFBS within a given distance (here 2 kb) of TSS and not weighting them depending on their distance or chromatin context.

The GO and IPA analysis of 787 genes mapped to high-confidence NF-κB/RelA-binding sites confirm established biological functions of NF-κB, including inflammation, anti-apoptosis and intracellular signaling pathways not yet fully described. We note, for example, that significant enrichment for genes encoding Rho, Ras and GTPase activity are detected (Supplementary Table S3). Here, oncogenic Ras activates NF-κB transcriptional activity through diverse mechanisms whose actions synergize in cellular transformation (39). Our finding suggests that NF-κB may also provide feedback control on Ras GTPase activity. This finding could be significant in the design of inhibitors of the Ras pathway, dysregulated in 30% of human tumors (40). Another novel biological function is that of cell motility and migration. In live-cell dynamic imaging studies, we have incidentally found that activation of NF-κB results in enhanced cellular motility [(41) and data not shown], thus confirming our computational finding. This data set thereby extends our knowledge of the cellular effects of NF-κB pathway activation.

Another interesting finding from our analysis is that the spectrum of GO molecular functions is different for genes with NF-κB/RelA binding in intronic sites, and for genes with NF-κB/RelA binding at different distances from the TSS. For example, NF-κB/RelA-binding sites are often located within 250 bp for NF-κB pathway genes and within 100 bp for base excision DNA repair. By contrast, NF-κB/RelA-binding sites are typically located within ±25 kb of genes involved in apoptosis and signaling (phosphorylation).

We performed analysis of statistically significant overlaps of our XChIP-Seq sites with ENCODE ChIP-Seq peaks for 119 TFs. As TFBSs have non-uniform distribution in the human genome, assessing whether TFBSs detected by ENCODE occur more frequently near NF-κB/RelA TFBSs than anywhere in the genome would not be appropriate. To correctly assess statistical significance of co-occurrence of NF-κB/RelA TFBSs with other TFBSs measured by ENCODE, we empirically estimated the *P*-value of such co-occurrence by comparing frequency of co-occurrence NF-κB/RelA TFBS with a given ENCODE TF versus its co-occurrence with all ENCODE TFs via 100 000 simulations per TF (algorithm described in detail in ‘Materials and Methods’ section). Results of these simulations (Table 3 and Supplementary Webpage) revealed exciting new insights into the nature of the chromatin domains, with NF-κB/RelA-AP1-enriched chromatin characteristically binding to repetitive regions that are reduced in phospho-Ser^2^-Pol II CTD binding, relatively reduced in DNase I hypersensitive sites, and are in DNA depleted in CpG islands, as compared with NF-κB/RelA-SP1 domains (Figure 8). Together these findings suggest that subnetworks of NF-κB-dependent genes can be identified that are distinct in co-associated TF sequence motifs, chromatin characteristics, location within structural regions of the gene and encode distinct biochemical mechanisms of activation. Consistent with these predictions is the temporal profile of NF-κB/RelA-AP1-regulated genes in our RNA-Seq experiments (Figure 7). Here, a distinct, slower profile of the NF-κB/RelA-AP1-regulated population is produced, perhaps consistent with the closed chromatin domain in which they are located. Our observation for inducible AP1 binding to the TANK promoter and its functional contribution to TANK mRNA expression is an experimental validation of our computational predictions.

NF-κB/RelA is a highly inducible transcription factor that functions in concert with other transcription factors including AP1. AP1 is a mitogen- and cytokine-inducible transcription factor that is activated by many of the same pathways that control NF-κB. There have been multiple levels of NF-κB-AP1 interaction that have been observed, including a functional interaction requiring binding of NF-κB and AP1 to composite binding sites in the regulatory regions of collagenase-3 (42) or co-adjacent sites as shown on the IL-8 (43) and RANTES (44) proximal promoters. Because these studies were focused analysis of individual promoters, any generalizations of the nature of their binding site interactions have not been possible. Here, we observe on a genome-wide level that AP1 binding is bimodally distributed and preferentially occurs at ∼100 nt distance from the NF-κB/RelA site. This spatial constraint may reflect the observation that RelA forms protein–protein complexes with AP1, thereby limiting the distance for functional interactions (45). Our statistical enrichment studies show that chromatin enriched for NF-κB/RelA-AP1 is distinct from that of NF-κB/RelA-SP1 chromatin (Table 4).

Our systematic *de novo* motif search strategy also has identified SP1 as an enriched motif associated with NF-κB/RelA enriched chromatin and the simulations using experimental ENCODE SP1 ChIP-Seq data confirmed this prediction (Table 3). Functional studies have shown that interactions between NF-κB and SP1 on target genes can be positive or inhibitory. Studies on the HIV LTR have shown that NF-κB-SP1 interactions promote DNA binding and transcriptional activity (46). SP1 is a transcription factor that mediates chromatin remodeling and gene regulation on TATA-less ‘GC-enriched’ gene promoters. Computational analyses have shown that the presence of a T inserted between the polyC submotifs (as in gapped and long gapped SP1 motifs) is an important determinant of the chromatin context in which such motif is found. The considerable variety in the length of SP1 motifs we uncovered suggests flexibility in SP1–DNA interaction (36). Although there is flexibility in the SP1 motif, the proximal bimodal distribution of SP1 sites relative to NF-κB/RelA also suggests that the interactions between these two *cis*-elements may be constrained by protein–protein interactions in *trans*. In contrast to the characteristics of the chromatin enriched in NF-κB/AP1 sites, our simulations using ENCODE data show that chromatin enriched in NF-κB/SP1 sites are more likely to be found in DNase I sensitive (open chromatin), associated with intronic locations in CpG islands and are associated with phospho-Ser^2^ RNA Pol II binding, as compared with NF-κB/SP1 sites. Recent work by our laboratory and others has shown that a subset of highly inducible NF-κB/RelA-dependent genes is regulated through a process of transcriptional elongation (14,47). This process is mediated in genes located in open chromatin domains by inducible cyclin-dependent kinase 9-mediated phosphorylation of Ser 2 RNA Pol II, resulting in the switch of RNA Pol II from a paused configuration into a highly processive mode (48,49). As a result, rapid gene induction is produced. Consistent with this mechanism, we observe the population of NF-κB/RelA-SP1-enriched genes are more rapidly induced (within 0.5 h) than the NF-κB/RelA-AP1 population (Figure 7).

A surprising finding from our *de novo* motif search studies has been the identification of enrichment of the ‘break point’ motif, a finding that would have been missed using a simpler, less computationally intensive method of verifying known DNA-binding motifs. The break point motif is a highly flexible DNA sequence associated with chromatin breaks (37), but it has not, to our knowledge, been shown to bind to any transcription factor. NF-κB/RelA binding to target genes produces histone acetylation, changes in nucleosomal rearrangement and inducing chromatin conformation through binding of co-activators and transcriptional elongation complexes to target genes (17,50–51). We speculate the enrichment of break point motif in NF-κB/RelA-binding sequences may indicate a requirement for chromatin flexibility induced by the biochemistry of NF-κB/RelA-dependent target gene expression. Moreover, this break point motif is present in one-third of cases within repetitive sequence, half of the times within Alu repeat. It is well known that such sequences have special properties with respect to DNA bendability.

On its activation, NF-κB induces the synthesis of inhibitors at distinct regulatory steps of the pathway. This NF-κB autoregulatory pathway thereby terminates NF-κB action so that the transcription factor is only transiently activated. The major inhibitors identified include those proteins with ankyrin repeat domains (IκB, BCL-3 and NF-κB-1,-2) responsible for binding and cytoplasmic redistribution of the NF-κB/RelA transcription factor itself, and deubiquitinases (TNFAIP3/A20 and CylD) whose actions are to remove Lys 63 linked polyubiquitin chains from the IKK complex terminating upstream signals. Our XChIP-Seq analysis has indicated that a previously unrecognized negative feedback inhibitor is TANK. TANK is a cytoplasmic protein that inhibits TRAF function by sequestering them in a latent state in the cytoplasm, and by recruiting a polo-like kinase that inhibits the ubiquitination of IKKγ and that of IKK activity (52). Our data show that TANK contains a cluster of NF-κB-binding sequences in its proximal promoter, and its mRNA is induced in a NF-κB/RelA-dependent mechanism, being sensitive to inhibition in the tetracycline-regulated cells (Figure 6). We suggest that the activation of negative feedback regulators (including TANK) maintains the cell in a refractory state after the NF-κB pathway has been activated.

Kinetic studies of NF-κB/RelA transcriptome have shown that the genomic response to NF-κB/RelA occurs in identifiable subnetworks of gene expression, with each wave encoding distinct biological pathways (11). Discussed earlier in the text, our computational findings suggest that, at least in part, kinetics of target activation may be due to distinct chromatin environments, with rapidly induced genes in an open chromatin configuration and delayed genes being located in a more closed chromatin where additional remodeling events would be required before gene activation (14,47). Moreover, our data indicate that the functional response of the NF-κB/RelA gene network is modified by the association of AP1- and SP1-binding sites at characteristic distances that segregate into distinct populations, where RelA-AP1-enriched chromatin is depleted in of RelA-SP1 motifs, and *vice versa* (Table 4). Consistent with our earlier finding using microarray studies, the biological pathways of genes enriched in rapidly responsive RelA-SP1 motifs control ‘signaling and gene expression’ and ‘programmed cell death’, whereas those in the slower-responding RelA-AP1 genes control ‘cytoskeleton/morphogenesis’ and ‘cellular migration/motility’. We interpret this to novel and interesting finding to suggest that the plethora of biological functions produced by NF-κB/RelA regulated genes may be a result of combination of different subpathways, each with a more restricted function whose precise timing of expression may be important for a coordinated biological response.

In conclusion, we propose a novel computational method that has enabled us to systematically analyze the human NF-κB cistrome and achieve highly accurate reconstruction of the NF-κB regulatory network. We identify the presence of enriched AP1, SP1 and break point motifs at characteristic distances from NF-κB/Rel A-binding domains. We discover how the co-occurrence of either SP1- or AP1-binding motifs with NF-κB/RelA are correlated with different chromatin configurations and temporal gene expression profiles in a biologically interpretable fashion. We suggest that the *cis* context of the NF-κB-binding site influences the kinetics and dynamic response of gene expression in the NF-κB network and sheds light on mechanism of action and function of temporally distinct subnetworks.

## ACCESSION NUMBERS

All the ChIP-Seq and RNA-Seq data from this study were deposited to NCBI Sequence Read Archive (http://www.ncbi.nlm.nih.gov/Traces/sra/sra.cgi) under accession number SRP020499.

## AVAILABILITY

Additional Supplementary Materials are also available from our supplementary webpage at: http://nfkb.utmb.edu.

## SUPPLEMENTARY DATA

Supplementary Data are available at NAR Online: Supplementary Tables 1–5 and Supplementary Figures 1–3.

## FUNDING

Multi-investigator Pilot Grant from the Sealy Center for Molecular Medicine (to A.R.B. and M.R.) (in part); National Institute of Health [PO1 AI062885 to A.R.B., NHLBI contract HHSN268201000037C to A.R.B., RO1 GM086885 (Kimmel, M. Rice University) UL1TR000071 UTMB CTSA to A.R.B.]; Polish Ministry of Science and Higher Education [N N519 652740 to N.D.]. Funding for open access charge: University Funds.

*Conflict of interest statement*. None declared.

## Supplementary Material

Supplementary Data
