# Supplementary material for: A probabilistic approach to learn chromatin architecture and accurate inference of the NF-κB/RelA regulatory network using ChIP-Seq
Source: Nucleic Acids Res. 2013 Jun 14;41(15):7240–59. doi: 10.1093/nar/gkt493 (PMC3753626; doi:10.1093/nar/gkt493)
Supplement: Supplementary Data [file supp_41_15_7240__index.html]

A probabilistic approach to learn chromatin architecture and accurate inference of the NF-κB/RelA regulatory network using ChIP-Seq — A probabilistic approach to learn chromatin architecture and accurate inference of the NF-κB/RelA regulatory network using ChIP-Seq — Supplementary Data 

# A probabilistic approach to learn chromatin architecture and accurate inference of the NF-κB/RelA regulatory network using ChIP-Seq

## Supplementary Data

files

**Files in this Data Supplement:**

- Supplementary Data - pdf file
