# Supplementary material for: A probabilistic approach to learn chromatin architecture and accurate inference of the NF-κB/RelA regulatory network using ChIP-Seq
Source: Nucleic Acids Res. 2013 Jun 14;41(15):7240–59. doi: 10.1093/nar/gkt493 (PMC3753626; doi:10.1093/nar/gkt493)
Supplement: Supplementary Data [file supp_gkt493_nar-01116-x-2013-File003.pdf]

Supplementary Table 1. ChIP-seq data quality. Shown are the total number of reads and those with Phred >= 20 that were 34 bp or longer, the number of unique reads (#U0 reads) mapped against the human genome assembly (GRCh37/hg19, Feb 2009) and percentage of total reads (U0%) in control and TNF stimulated samples. Among the samples, Con and TNF were single-end sequenced on an Illumina Genome Analyzer II for 40 cycles, while samples of Con_rep and TNF_rep were from an independent replication ChIP experiment and were sequenced on an Illumina HiSeq 1000 for 50 cycles.

Samples	Total # reads	Phred >=20; Len >= 34	#U0 reads	U0 %	% mappable	% poly-A
**Con**	22,492,088	17,862,626	13,805,022	77	88	6
**TNF**	32,563,372	12,624,879	10,212,535	81	91	4
**Con_rep**	84,708,370	69,415,853	54,089,356	78	86	2
**TNF_rep**	73,906,730	60,850,095	48,900,736	80	88	2

Supplementary Table 2. Novel RelA peaks for qualitative validation. All the MACS identified peaks were rank-ordered based on the MACS quality scores. 21 novel regions were selected for qualitative validation, 11 from the top of the rank-ordered peak list and 10 from the bottom of the peak list. The detailed information of a total of 21 peaks is listed in the table below, including its chromosome #, position, peak lable, significance score and the forward and reverse primer sequences used for Q-gPCR validation.

Chr #	Start Position	End Position	RelA Peak Label	Significance Score	Forward primer	Reverse primer
chr10	30721275	30724170	peak_16	1149.15	GCGAGTACACTAAGATATACGA	TGACCCAAGGCGTCTTAG
chr1	204380162	204381921	peak_154	705.71	ATGCGCAGTCGTTTCTGTGT	CATCAAAACGCCTCCGGGAA
chr19	39389461	39391628	peak_278	613.28	CTAGAGAGTTGTAGTCCT	AATGATAATATGCTGAATCG
chr4	76438297	76439601	peak_439	537.87	GAACGGAAAACCCCAGTA	TTTCCTACCTGCTTCGTA
chr1	156023710	156024811	peak_582	493.51	CTTGGAGGTTGGGTTTTC	CGTTCTTAATAGACCATTCAG
chr1	23693868	23695040	peak_856	437.41	GCCAGCGAATTACTAATCC	ACCTTGTCTCTGCTTACC
chr2	55458814	55460999	peak_1024	412.1	AAGTTCACGTCCTAGTCTGG	CTTAGGCGACCACACCAA
chr17	37617179	37618155	peak_1162	390.65	CGTTTCTAGTTTGGGACCTGAT	GATGTTTCGTGCTTCGCTTT
chr2	161992816	161994168	peak_1207	384.14	GAAGTCAGAGCTACAGTCA	CCAATCATGGCACGATAC
chr2	208392586	208395088	peak_1510	346.45	TTTAGGGTGCAGTGGGTG	CCTTTCTCCAGACAACATCCT
chr19	49374169	49377163	peak_3929	208.2	GCATTTGATTGACAGTTC	GAAGTGGAAGTAAGACAG
chr6	39067167	39067966	peak_20572	50.4	TGTCATTTCACTTCTCAGAT	TCCTCATATCCTCACCAA
chr8	54672219	54673553	peak_20573	50.4	GACACTGAACATGACAGC	TTATACATTGGCATAGTTCCG
chr16	57657194	57658465	peak_20577	50.39	GGTCTGAGATCAGGTCCT	GTTCCCATTTAACAGAGATGAAA
chr1	23911537	23912922	peak_20600	50.34	AATCTAGACAAGTTCTGAGAC	AGAAGGACGCTCTGTAAA
chr10	102105326	102106115	peak_20608	50.32	AAATCCCAGGTGCTTGTC	TTTCCCAAATACTGCCCTA
chr1	84687772	84688574	peak_20646	50.22	TCCAGATGTAGGACTACC	TCTTTCCCAGATAAGCAAA
chr18	10223369	10224171	peak_20647	50.22	CATTCTCCATTTACCTGTTT	GACACATAGCTCCAACAT
chr15	70042528	70043344	peak_20663	50.19	GCAATCAAAGTGTTTACTGA	AACCAACAACTTGAGTGA
chr18	3162137	3162833	peak_20684	50.13	TGAACTAGCCAATCCTAACT	CCTAGACCAAGCCCTTTA
chrX	47076417	47077800	peak_20699	50.07	GCAAGAGAGGTGGCAGGAA	GCGGAGCCTCTTTTCTGC

Supplementary Table 3. Genome ontology. Shown are the p-value for enrichment, the number of genes relative to the total genes in the GO category, and the GO term (Abbreviations: NOD, nucleotide-binding oligomerization domain.).

**Term**	**Biological Process**	***q-value***	**Count**
GO:0042981	regulation of apoptosis	1.14E-09	78
GO:0043067	regulation of programmed cell death	9.34E-10	78
GO:0010941	regulation of cell death	7.74E-10	78
GO:0048522	positive regulation of cellular process	2.58E-07	127
GO:0048518	positive regulation of biological process	9.26E-07	134
GO:0006950	response to stress	9.26E-06	113
GO:0006915	apoptosis	4.47E-05	53
GO:0012501	programmed cell death	6.24E-05	53
GO:0016265	death	8.26E-05	59
GO:0006357	regulation of transcription from RNA polymerase II promoter	8.45E-05	59
GO:0009605	response to external stimulus	9.65E-05	69
GO:0031325	positive regulation of cellular metabolic process	9.89E-05	67
GO:0008219	cell death	1.02E-04	58
GO:0009893	positive regulation of metabolic process	9.88E-05	69
GO:0043065	positive regulation of apoptosis	9.97E-05	41
GO:0043068	positive regulation of programmed cell death	1.11E-04	41
GO:0010942	positive regulation of cell death	1.18E-04	41
GO:0010604	positive regulation of macromolecule metabolic process	1.16E-04	65
GO:0009891	positive regulation of biosynthetic process	1.19E-04	56
GO:0031328	positive regulation of cellular biosynthetic process	1.65E-04	55
GO:0045944	positive regulation of transcription from RNA polymerase II promoter	2.68E-04	36
GO:0051173	positive regulation of nitrogen compound metabolic process	2.62E-04	52
GO:0010646	regulation of cell communication	5.42E-04	72
GO:0006917	induction of apoptosis	5.24E-04	32
GO:0012502	induction of programmed cell death	5.36E-04	32
GO:0043122	regulation of I-kappaB kinase/NF-kappaB cascade	5.17E-04	17
GO:0010557	positive regulation of macromolecule biosynthetic process	7.26E-04	51
GO:0006916	anti-apoptosis	7.84E-04	24
GO:0042127	regulation of cell proliferation	7.68E-04	58
GO:0007242	intracellular signaling cascade	7.82E-04	82
GO:0045935	positive regulation of nucleobase, nucleoside, nucleotide and nucleic acid metabolic	8.15E-04	49
GO:0043066	negative regulation of apoptosis	0.001163684	33
GO:0045893	positive regulation of transcription, DNA-dependent	0.001398436	40
GO:0009611	response to wounding	0.001375248	43
GO:0042221	response to chemical stimulus	0.001373837	82
GO:0043069	negative regulation of programmed cell death	0.001373638	33
GO:0065007	biological regulation	0.001404282	347
GO:0060548	negative regulation of cell death	0.001375397	33
GO:0051254	positive regulation of RNA metabolic process	0.00142974	40
GO:0010627	regulation of protein kinase cascade	0.001438052	26
GO:0009966	regulation of signal transduction	0.001811348	61
GO:0043123	positive regulation of I-kappaB kinase/NF-kappaB cascade	0.001876827	15
GO:0010628	positive regulation of gene expression	0.002024875	45
GO:0001775	cell activation	0.001980048	28
GO:0045321	leukocyte activation	0.002276207	25
GO:0010740	positive regulation of protein kinase cascade	0.002231826	20

GO:0006954	inflammatory response	0.002536551	30
GO:0048583	regulation of response to stimulus	0.00284779	38
GO:0002376	immune system process	0.003037295	66
GO:0002366	leukocyte activation during immune response	0.003747911	9
GO:0002263	cell activation during immune response	0.003747911	9
GO:0045941	positive regulation of transcription	0.003835478	43
GO:0007243	protein kinase cascade	0.004161903	32
GO:0050789	regulation of biological process	0.004419499	328
GO:0050794	regulation of cellular process	0.007535828	315
GO:0048519	negative regulation of biological process	0.007495622	103
GO:0009607	response to biotic stimulus	0.007665026	32
GO:0010033	response to organic substance	0.008413938	50
GO:0009987	cellular process	0.009442406	456
GO:0002250	adaptive immune response	0.01038834	12
GO:0002460	adaptive immune response based on somatic recombination of immune receptors bu	0.01038834	12
GO:0006952	defense response	0.010881025	44
GO:0070431	nucleotide-binding oligomerization domain containing 2 signaling pathway	0.011017349	4
GO:0070423	nucleotide-binding oligomerization domain containing signaling pathway	0.011017349	4
GO:0043331	response to dsRNA	0.015945454	7
GO:0031323	regulation of cellular metabolic process	0.015765779	174
GO:0048523	negative regulation of cellular process	0.015926638	94
GO:0065008	regulation of biological quality	0.016425332	85
GO:0008624	induction of apoptosis by extracellular signals	0.020977903	14
GO:0009991	response to extracellular stimulus	0.020916987	21
GO:0002285	lymphocyte activation during immune response	0.022056187	6
GO:0051259	protein oligomerization	0.023189712	18
GO:0002443	leukocyte mediated immunity	0.023079086	12
GO:0052547	regulation of peptidase activity	0.023079086	12
GO:0031663	lipopolysaccharide-mediated signaling pathway	0.025299175	5
GO:0043330	response to exogenous dsRNA	0.025299175	5
GO:0010647	positive regulation of cell communication	0.026817043	27
GO:0050776	regulation of immune response	0.026881398	21
GO:0060255	regulation of macromolecule metabolic process	0.028704856	163
GO:0007249	I-kappaB kinase/NF-kappaB cascade	0.029821979	10
GO:0050793	regulation of developmental process	0.030683509	45
GO:0019222	regulation of metabolic process	0.031010729	178
GO:0031667	response to nutrient levels	0.031528015	19
GO:0002252	immune effector process	0.031275611	15
GO:0046649	lymphocyte activation	0.034497884	19
GO:0051789	response to protein stimulus	0.037435602	13
GO:0034097	response to cytokine stimulus	0.037289456	11
GO:0043281	regulation of caspase activity	0.037289456	11
GO:0048584	positive regulation of response to stimulus	0.040116761	21
GO:0002253	activation of immune response	0.040437314	12
GO:0010556	regulation of macromolecule biosynthetic process	0.040277053	143
GO:0008283	cell proliferation	0.041869948	32
GO:0002286	T cell activation during immune response	0.041638864	5
GO:0006955	immune response	0.042598249	45
GO:0001933	negative regulation of protein amino acid phosphorylation	0.04423912	7
GO:0052548	regulation of endopeptidase activity	0.045186542	11
GO:0050896	response to stimulus	0.048448966	171

Term	Cellular Component	*q-value*	Count
GO:0005737	cytoplasm	1.19E-08	364
GO:0005622	intracellular	9.37E-07	489
GO:0005829	cytosol	3.43E-06	93
GO:0044424	intracellular part	7.17E-06	471
GO:0005634	nucleus	0.001234922	246
GO:0044444	cytoplasmic part	0.001260871	238
GO:0043231	intracellular membrane-bounded organelle	0.00118921	360
GO:0043227	membrane-bounded organelle	0.001145517	360
GO:0043229	intracellular organelle	0.009327281	391
GO:0033256	I-kappaB/NF-kappaB complex	0.009561233	4
GO:0043226	organelle	0.008817434	391

Term	Molecular Function	*q-value*	Count
GO:0005515	protein binding	5.09E-09	404
GO:0017048	Rho GTPase binding	6.02E-04	11
GO:0017016	Ras GTPase binding	0.003363176	15
GO:0008134	transcription factor binding	0.003070999	42
GO:0019899	enzyme binding	0.00385232	42
GO:0051020	GTPase binding	0.00328871	16
GO:0005488	binding	0.003230931	532
GO:0031267	small GTPase binding	0.004209836	15
GO:0030528	transcription regulator activity	0.017929702	88

Supplementary Figure 1. Weblogos found in *ab initio* search. 1A. Short and long versions of major motifs found in the top 20% of NF-κB/RelA MACS peaks. 1B. Four variants of the Sp1 motif, showing that the gaps between C residues can vary substantially.

1A

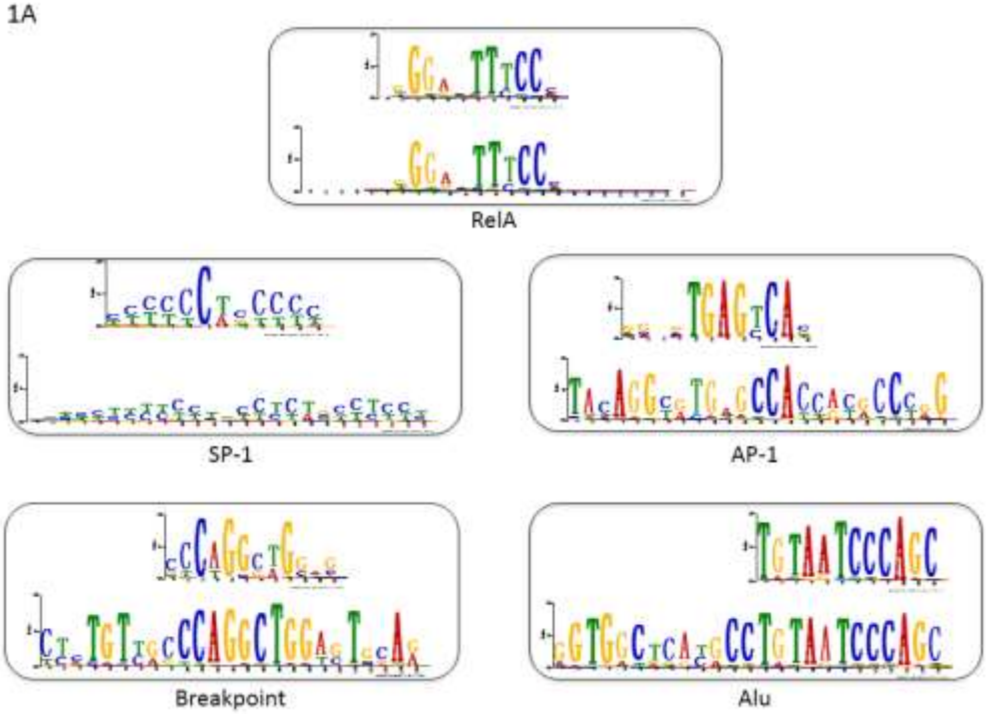

1B

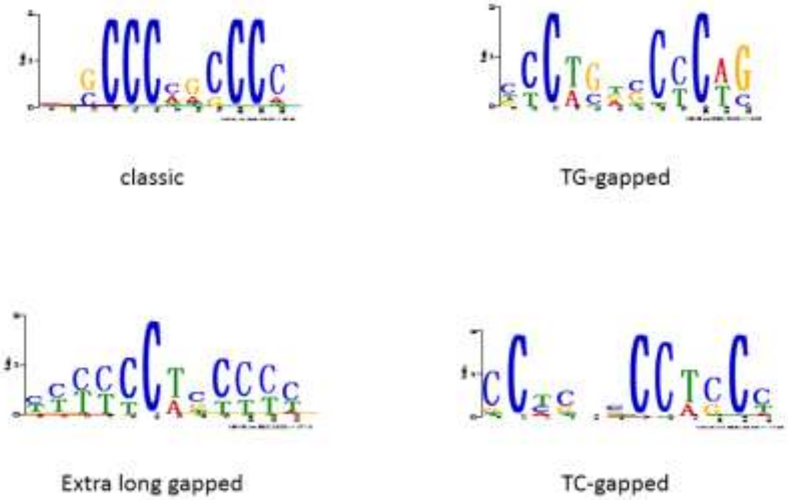

Supplementary Figure 2. Comparison of Informativity scores of a top cutoff list of genes derived by the scored probabilistic approach and the naïve nearest gene approach. The genes from the scored probabilistic approach score much higher on the Informativity scores and thus have greater biological significances. The following charts show the Informativity scores of the given list compared to Informativity scores of 10 random list of genes.

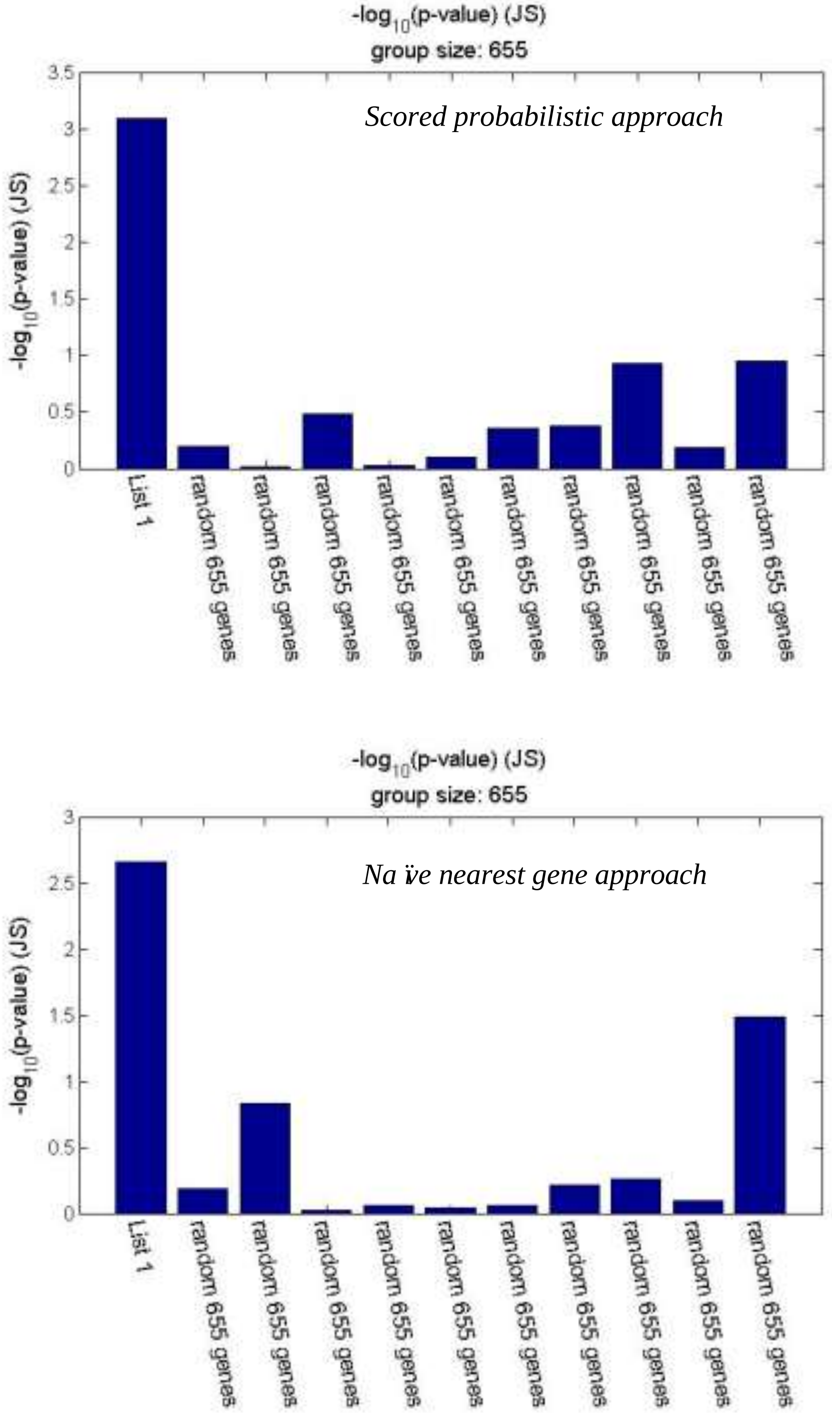

Supplementary Figure 3. Screening of c-Jun shRNAs. A549 cells were transduced with lentiviral c-Jun shRNAs and selected with puromycin for stable gene knockdown. c-Jun mRNA was analyzed by real-time PCR using human c-Jun specific primers, forward, 5'-GCATCATCTGTAGATACTA-3'; reverse, 5'-GGTAAGCAATTCCATATAG-3'. The result suggested that TRCN0000355646 (*) can efficiently knock down c-Jun expression in A549 cells and was used in the experiments with pLKO.1-puro as a negative control.

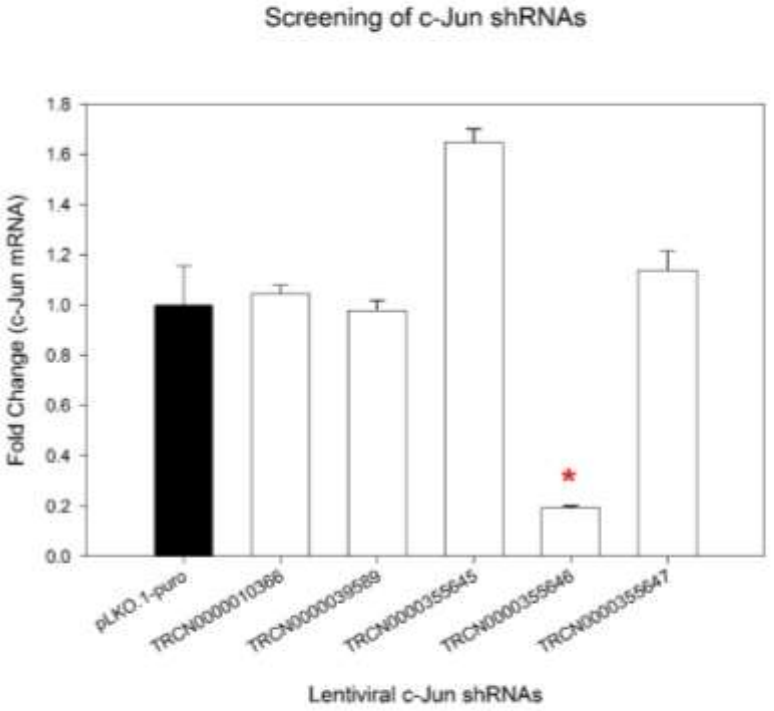

Supplementary Table 4. Ordered list of genes scored by their sum of odds of regulation. The list contains top 500 genes.

GeneName	Score (sum of odds)	ID
PDE4D	38.612	ENSG00000113448.11
**TANK**	28.8329	ENSG00000136560.8
REPIN1	26.5998	ENSG00000214022.6
PLEC	25.7768	ENSG00000178209.10
NCOA7	24.6911	ENSG00000111912.13
ZFP36L1	24.5167	ENSG00000185650.7
TMEM75	23.8932	ENSG00000256655.1
RNF19A	22.4454	ENSG00000034677.7
MAP3K8	22.124	ENSG00000107968.4
TNIP1	21.9417	ENSG00000145901.9
ST5	21.8525	ENSG00000166444.12
SOX9	21.5062	ENSG00000125398.4
SLC39A11	21.2379	ENSG00000133195.6
NFE2L2	20.5946	ENSG00000116044.10
MAFF	20.5232	ENSG00000185022.7
CLLU1	20.3538	ENSG00000257127.1
OLA1	20.0932	ENSG00000138430.11
ALCAM	20.0724	ENSG00000170017.8
RBKS	19.8472	ENSG00000171174.9
MYC	19.8307	ENSG00000136997.9
GRHL2	19.6823	ENSG00000083307.5
NEK6	19.4567	ENSG00000119408.11
SQSTM1	18.9651	ENSG00000161011.14
PTHLH	18.8315	ENSG00000087494.10
SPAG1	18.7234	ENSG00000104450.8
TMTC3	18.7234	ENSG00000139324.7
ABCA5	18.7044	ENSG00000154265.10
ELF3	18.6341	ENSG00000163435.10
CXCL1	18.597	ENSG00000163739.4
AKR1C2	18.2527	ENSG00000151632.11
TP63	18.0864	ENSG00000073282.7
MYADM	17.9886	ENSG00000179820.10
TLE1	17.644	ENSG00000196781.8
NR4A2	17.5822	ENSG00000153234.9
BOD1	17.3487	ENSG00000145919.6
C8orf4	17.1063	ENSG00000176907.3
FAM65C	16.9812	ENSG00000042062.7
NAMPT	16.8719	ENSG00000105835.7
TNFAIP3	16.8256	ENSG00000118503.9
BCL3	16.7496	ENSG00000069399.7
ITGB1	16.629	ENSG00000150093.13
EFR3A	16.6091	ENSG00000132294.8

TNFSF15	16.5798	ENSG00000181634.6
GFRA1	16.5437	ENSG00000151892.9
SH3BP4	16.4366	ENSG00000130147.10
ZMAT4	16.2408	ENSG00000165061.10
RAB3C	16.227	ENSG00000152932.6
DUSP10	16.2187	ENSG00000143507.12
JAK1	16.1488	ENSG00000162434.7
OLIG3	15.9997	ENSG00000177468.5
NR4A1	15.9989	ENSG00000123358.14
ZBTB25	15.9476	ENSG00000089775.6
DDR1	15.9049	ENSG00000204580.6
SP3	15.8933	ENSG00000172845.9
TPBG	15.7419	ENSG00000146242.5
CREB1	15.6195	ENSG00000118260.8
ADAM2	15.5526	ENSG00000104755.10
BRE	15.3974	ENSG00000158019.14
RBMS1	15.3196	ENSG00000153250.13
MKLN1	15.3172	ENSG00000128585.11
ADTRP	15.2848	ENSG00000111863.7
CSF1	15.2796	ENSG00000184371.9
SEPT9	15.2504	ENSG00000184640.10
KCNS3	15.2205	ENSG00000170745.7
CDK17	14.9964	ENSG00000059758.3
BDKRB2	14.9932	ENSG00000168398.5
ZNF296	14.9915	ENSG00000170684.2
SLC35D3	14.9787	ENSG00000182747.4
ARHGAP26	14.8942	ENSG00000145819.10
CXXC5	14.8731	ENSG00000171604.7
AAGAB	14.8424	ENSG00000103591.8
CD36	14.8378	ENSG00000135218.13
GRAMD1A	14.7941	ENSG00000089351.7
NFKBIA	14.793	ENSG00000100906.6
MARCH7	14.6968	ENSG00000136536.9
PARD3	14.495	ENSG00000148498.11
STAT1	14.468	ENSG00000115415.13
BHLHE40	14.4295	ENSG00000134107.4
AC109486.1	14.3946	ENSG00000224838.2
SSTR2	14.3946	ENSG00000180616.3
AKR1B1	14.1969	ENSG00000085662.8
ZNF706	14.1969	ENSG00000120963.7
PLXNB2	14.1324	ENSG00000196576.9
TLCD1	14.1324	ENSG00000160606.5
PRKCH	14.1272	ENSG00000027075.9
CEACAM16	14.0716	ENSG00000213892.4
SLC25A45	14.0525	ENSG00000162241.8
SLC11A2	14.0452	ENSG00000110911.10

KLF6	14.0355	ENSG00000067082.9
ANKRD18B	14.0169	ENSG00000230453.3
PCSK2	13.9722	ENSG00000125851.5
C9orf3	13.8728	ENSG00000148120.9
PIK3C2B	13.8495	ENSG00000133056.8
C11orf82	13.8313	ENSG00000165490.8
FAM84B	13.7992	ENSG00000168672.3
PTGS2	13.7965	ENSG00000073756.7
COL4A2	13.7562	ENSG00000134871.12
DEC1	13.6563	ENSG00000173077.10
KCNMA1	13.6475	ENSG00000156113.13
RAD51B	13.6356	ENSG00000182185.12
SULT2B1	13.5893	ENSG00000088002.6
RPH3AL	13.5711	ENSG00000181031.11
STAT6	13.5711	ENSG00000166888.4
SVIL	13.5313	ENSG00000197321.8
FCHSD2	13.5089	ENSG00000137478.9
TXNRD1	13.4955	ENSG00000198431.10
AC021066.1	13.4599	ENSG00000170442.6
DRD1	13.4441	ENSG00000184845.2
TRMT61B	13.4441	ENSG00000171103.6
PERP	13.4172	ENSG00000112378.9
GPBP1	13.4164	ENSG00000062194.10
KCNJ15	13.3469	ENSG00000157551.12
SLC9A8	13.341	ENSG00000197818.7
EXD2	13.2873	ENSG00000081177.12
GPRC5A	13.2235	ENSG00000013588.5
ST3GAL4	13.2215	ENSG00000110080.13
MAML2	13.2187	ENSG00000184384.8
ZNF180	13.2031	ENSG00000167384.5
RPL23A	13.154	ENSG00000198242.7
FAM174B	13.1461	ENSG00000185442.6
LOX	13.1461	ENSG00000113083.7
SOX13	13.1148	ENSG00000143842.10
EPS8	13.0697	ENSG00000151491.7
MID1	13.0197	ENSG00000101871.8
NAV1	12.9616	ENSG00000134369.10
RAPH1	12.9476	ENSG00000173166.12
TPRA1	12.9207	ENSG00000163870.10
ECHDC1	12.9051	ENSG00000093144.13
AKR1C1	12.8538	ENSG00000187134.8
KTN1	12.8514	ENSG00000126777.13
CBLB	12.848	ENSG00000114423.14
IER3	12.8209	ENSG00000137331.10
CRYAA	12.7467	ENSG00000160202.3
GRB7	12.724	ENSG00000141738.8

PSEN1	12.724	ENSG00000080815.13
TNS3	12.6943	ENSG00000136205.11
IL8	12.6898	ENSG00000169429.6
ALPK2	12.6497	ENSG00000198796.5
DYRK2	12.613	ENSG00000127334.10
DNAJB6	12.5831	ENSG00000105993.9
ARL4C	12.5499	ENSG00000188042.5
LMOD1	12.5499	ENSG00000163431.10
CXCL2	12.5347	ENSG00000081041.7
ACHE	12.4978	ENSG00000087085.8
CDC42EP4	12.4978	ENSG00000179604.7
TP53	12.4978	ENSG00000141510.9
UXS1	12.4978	ENSG00000115652.10
LEPREL1	12.4929	ENSG00000090530.5
OCLM	12.4823	ENSG00000262180.1
MYO16	12.4732	ENSG00000041515.9
BCAR1	12.4709	ENSG00000050820.12
CD63	12.4709	ENSG00000135404.6
GEM	12.4709	ENSG00000164949.3
SHB	12.469	ENSG00000107338.8
PTPN1	12.44	ENSG00000196396.5
NEDD4L	12.4297	ENSG00000049759.11
C10orf11	12.4182	ENSG00000148655.8
ARHGAP27	12.3817	ENSG00000159314.5
MTHFD2L	12.3758	ENSG00000163738.13
ITGB8	12.3747	ENSG00000105855.4
SYNPO	12.3546	ENSG00000171992.8
ISM1	12.3298	ENSG00000101230.4
DPP4	12.2945	ENSG00000197635.5
PDE6D	12.2945	ENSG00000156973.9
CLIP2	12.2884	ENSG00000106665.10
RPS27A	12.2791	ENSG00000143947.8
LAMB3	12.2326	ENSG00000196878.7
AL137145.1	12.2004	ENSG00000212743.1
DLX2	12.1979	ENSG00000115844.6
GRM3	12.18	ENSG00000198822.6
KLF4	12.1454	ENSG00000136826.9
PFKP	12.1349	ENSG00000067057.10
LCTL	12.1028	ENSG00000188501.7
TBC1D22A	11.9849	ENSG00000054611.9
ALDH3A1	11.9588	ENSG00000108602.12
S100A10	11.9588	ENSG00000197747.4
ITIH2	11.9517	ENSG00000151655.12
C2	11.912	ENSG00000166278.9
CXCL3	11.8204	ENSG00000163734.4
SYT2	11.8204	ENSG00000143858.7

SMAD3	11.8145	ENSG00000166949.10
LMNA	11.7576	ENSG00000160789.14
MARCKS	11.7476	ENSG00000155130.5
PHLDB2	11.735	ENSG00000144824.14
CXorf36	11.6983	ENSG00000147113.12
PLLP	11.6983	ENSG00000102934.5
SIAH1	11.6714	ENSG00000196470.7
SH3PXD2A	11.659	ENSG00000107957.10
SHROOM3	11.6491	ENSG00000138771.9
CD34	11.6444	ENSG00000174059.11
GADD45B	11.6292	ENSG00000099860.3
SIK1	11.621	ENSG00000142178.7
PAPPA	11.6066	ENSG00000182752.7
PRKCQ	11.5987	ENSG00000065675.9
MUC2	11.5825	ENSG00000198788.7
TNS4	11.5592	ENSG00000131746.7
DUSP6	11.5466	ENSG00000139318.6
GPX4	11.5057	ENSG00000167468.10
PTP4A3	11.5056	ENSG00000184489.7
ZEB1	11.4956	ENSG00000148516.15
IST1	11.4758	ENSG00000182149.13
LCN2	11.4758	ENSG00000148346.7
NCOA4	11.4758	ENSG00000138293.14
SKIL	11.4758	ENSG00000136603.9
AKR1E2	11.4436	ENSG00000165568.12
CFLAR	11.4165	ENSG00000003402.12
LZIC	11.3976	ENSG00000162441.6
SS18	11.3976	ENSG00000141380.8
TMBIM1	11.3976	ENSG00000135926.6
UGT2A3	11.3976	ENSG00000135220.6
RIPK2	11.3648	ENSG00000104312.6
SETD9	11.3547	ENSG00000155542.6
PDE4B	11.3469	ENSG00000184588.13
WWC1	11.3109	ENSG00000113645.8
AL139147.1	11.2436	ENSG00000248458.2
C10orf68	11.234	ENSG00000150076.16
MTA3	11.234	ENSG00000057935.8
DDIT4	11.2238	ENSG00000168209.3
WDR11	11.2041	ENSG00000120008.10
ETS2	11.1866	ENSG00000157557.7
CERK	11.1739	ENSG00000100422.9
CASP10	11.1718	ENSG00000003400.10
HDDC2	11.1516	ENSG00000111906.12
TNFAIP8	11.1495	ENSG00000145779.6
CTSB	11.1324	ENSG00000164733.14
GPR108	11.1324	ENSG00000125734.7

IFT20	11.1324	ENSG00000109083.8
LBR	11.1324	ENSG00000143815.10
AKAP13	11.1264	ENSG00000170776.14
IRS2	11.0946	ENSG00000185950.7
PODXL	11.0946	ENSG00000128567.11
TBC1D7	11.0946	ENSG00000145979.11
PFDN4	11.0829	ENSG00000101132.5
PTMA	11.0829	ENSG00000187514.9
PLA2G4A	11.0728	ENSG00000116711.7
CA12	11.0707	ENSG00000074410.9
PLEKHG2	11.0556	ENSG00000090924.8
ABI1	11.0452	ENSG00000136754.12
C1orf132	11.0452	ENSG00000203709.4
KIAA0430	11.0452	ENSG00000166783.13
NRXN2	11.0452	ENSG00000110076.13
BCL6	11.0026	ENSG00000113916.13
ABHD2	10.9726	ENSG00000140526.12
IDE	10.971	ENSG00000119912.10
ATG2B	10.9455	ENSG00000066739.7
DUSP5	10.9419	ENSG00000138166.4
AP1S3	10.8961	ENSG00000152056.12
GALNTL4	10.8961	ENSG00000110328.5
CCL20	10.779	ENSG00000115009.7
PLEKHH2	10.7689	ENSG00000152527.9
SAR1B	10.759	ENSG00000152700.8
DICER1	10.7517	ENSG00000100697.9
TBL1XR1	10.7471	ENSG00000177565.10
TPD52	10.7471	ENSG00000076554.9
TRIB1	10.7251	ENSG00000173334.3
AMOTL2	10.7201	ENSG00000114019.10
HNRNPF	10.7201	ENSG00000169813.11
RELA	10.7201	ENSG00000173039.12
SH2D3A	10.7201	ENSG00000125731.7
SLC24A6	10.7201	ENSG00000089060.6
TMEM170B	10.6702	ENSG00000205269.4
ALDH1A1	10.6475	ENSG00000165092.7
MIER3	10.6475	ENSG00000155545.14
RPS27L	10.6475	ENSG00000185088.8
TCF7L2	10.6403	ENSG00000148737.11
FOSL1	10.6204	ENSG00000175592.3
PPP1R15B	10.6204	ENSG00000158615.7
RHCG	10.6204	ENSG00000140519.7
SLC8A1	10.6126	ENSG00000183023.12
GPR37L1	10.606	ENSG00000170075.6
JUN	10.5981	ENSG00000177606.5
MTMR2	10.5981	ENSG00000087053.12

BBC3	10.5711	ENSG00000105327.10
ITPRIP	10.5711	ENSG00000148841.10
RARG	10.5711	ENSG00000172819.11
DUSP1	10.5587	ENSG00000120129.4
EGR1	10.5219	ENSG00000120738.6
PLAU	10.5219	ENSG00000122861.9
COLEC10	10.4993	ENSG00000184374.2
NR3C1	10.4984	ENSG00000113580.9
TFEC	10.4984	ENSG00000105967.11
MTPAP	10.4366	ENSG00000107951.7
CMIP	10.4319	ENSG00000153815.9
LIMCH1	10.369	ENSG00000064042.12
KIRREL3	10.3494	ENSG00000149571.6
PTGER4	10.3363	ENSG00000171522.5
DENND3	10.3045	ENSG00000105339.6
AJUBA	10.2973	ENSG00000129474.11
C1orf63	10.2973	ENSG00000117616.12
C7orf23	10.2973	ENSG00000135185.7
CBX5	10.2973	ENSG00000094916.8
CHST15	10.2973	ENSG00000182022.12
CLIC1	10.2973	ENSG00000213719.4
CYB5A	10.2973	ENSG00000166347.12
EIF2AK3	10.2973	ENSG00000172071.6
GABPB1	10.2973	ENSG00000104064.11
HARBI1	10.2973	ENSG00000180423.4
IGSF3	10.2973	ENSG00000143061.13
KLK10	10.2973	ENSG00000129451.6
LPXN	10.2973	ENSG00000110031.8
MCM7	10.2973	ENSG00000166508.12
NIPA1	10.2973	ENSG00000170113.11
PAN2	10.2973	ENSG00000135473.10
PRCP	10.2973	ENSG00000137509.5
RBM23	10.2973	ENSG00000100461.12
S100A2	10.2973	ENSG00000196754.5
S100A3	10.2973	ENSG00000188015.5
SLC16A1	10.2973	ENSG00000155380.7
SNUPN	10.2973	ENSG00000169371.9
UNKL	10.2973	ENSG00000059145.13
ZCCHC8	10.2973	ENSG00000033030.9
ZFHX2	10.2973	ENSG00000136367.9
CPLX2	10.271	ENSG00000145920.9
BNIP1	10.2575	ENSG00000113734.13
PDZD2	10.2575	ENSG00000133401.10
AP001496.1	10.2474	ENSG00000228344.1
RUSC1	10.2055	ENSG00000160753.11
STAT5A	10.2055	ENSG00000126561.9

DKK3	10.2004	ENSG00000050165.12
TMEM30B	10.2004	ENSG00000182107.5
NEDD9	10.1734	ENSG00000111859.12
GNA12	10.1465	ENSG00000146535.8
FGD6	10.1195	ENSG00000180263.9
ZHX2	10.1165	ENSG00000178764.6
GLIS3	10.1085	ENSG00000107249.15
RAB1A	10.0815	ENSG00000138069.12
SBNO2	10.069	ENSG00000064932.9
MASTL	10.0645	ENSG00000120539.9
UBB	10.0645	ENSG00000170315.6
ZBTB38	10.0626	ENSG00000177311.6
ITSN2	10.0591	ENSG00000198399.8
JAG1	10.0514	ENSG00000101384.6
BFSP1	10.0111	ENSG00000125864.7
ALKBH4	9.99432	ENSG00000160993.3
ARL8A	9.99432	ENSG00000143862.3
C22orf34	9.99432	ENSG00000188511.8
GBX2	9.99432	ENSG00000168505.6
HDAC2	9.99432	ENSG00000196591.7
TASP1	9.99432	ENSG00000089123.10
DCAF10	9.98582	ENSG00000122741.11
HMBOX1	9.98582	ENSG00000147421.12
HORMAD2	9.98582	ENSG00000176635.12
IGFBP1	9.98582	ENSG00000146678.5
CREM	9.96548	ENSG00000095794.14
GOLGA7	9.96548	ENSG00000147533.11
AOAH	9.95947	ENSG00000136250.6
TGIF1	9.93381	ENSG00000177426.14
ITGB6	9.92463	ENSG00000115221.6
IGF2BP1	9.91235	ENSG00000159217.5
CARD14	9.91014	ENSG00000141527.10
GCNT3	9.91014	ENSG00000140297.8
PLEKHG6	9.85084	ENSG00000008323.11
ARL5B	9.83465	ENSG00000165997.4
NFKB1	9.83465	ENSG00000109320.6
DNAH7	9.79588	ENSG00000118997.8
FBXW11	9.79588	ENSG00000072803.13
PWWP2A	9.79588	ENSG00000170234.7
SBF2	9.78348	ENSG00000133812.10
ZNRF1	9.7714	ENSG00000186187.6
TSKU	9.71846	ENSG00000182704.5
ANKRD46	9.70087	ENSG00000186106.7
ZFP36L2	9.69625	ENSG00000152518.5
SLC39A10	9.67397	ENSG00000196950.9
HES1	9.67176	ENSG00000114315.3

ABTB2	9.66929	ENSG00000166016.4
IL31RA	9.65004	ENSG00000164509.9
ASAP1	9.64685	ENSG00000153317.9
SHC3	9.64685	ENSG00000148082.5
ZBED3	9.64685	ENSG00000132846.5
FARS2	9.63982	ENSG00000145982.5
SPECC1	9.63982	ENSG00000128487.10
B4GALT5	9.60155	ENSG00000158470.5
IRF2BP2	9.60155	ENSG00000168264.6
MKL2	9.58584	ENSG00000186260.11
METTL15	9.56296	ENSG00000169519.14
ERG	9.54722	ENSG00000157554.14
NKIRAS1	9.54722	ENSG00000197885.6
FOSL2	9.53302	ENSG00000075426.7
C15orf52	9.50243	ENSG00000188549.7
DENND4A	9.49781	ENSG00000174485.9
SLC25A12	9.49781	ENSG00000115840.9
IKBKE	9.48603	ENSG00000143466.7
MAP2K3	9.48603	ENSG00000034152.13
SLC25A37	9.48603	ENSG00000147454.8
C1orf86	9.47085	ENSG00000162585.11
CD59	9.47085	ENSG00000085063.10
CDC42EP5	9.47085	ENSG00000167617.2
CSAD	9.47085	ENSG00000139631.12
DLG4	9.47085	ENSG00000132535.11
FLNA	9.47085	ENSG00000196924.9
HAVCR1	9.47085	ENSG00000113249.8
MAFG	9.47085	ENSG00000197063.6
RAPGEF3	9.47085	ENSG00000079337.10
SLC7A5	9.47085	ENSG00000103257.4
STK40	9.47085	ENSG00000196182.6
USP54	9.47085	ENSG00000166348.12
ARNT2	9.46756	ENSG00000172379.13
TACC2	9.46682	ENSG00000138162.11
EHBP1	9.445	ENSG00000115504.9
BMP6	9.42201	ENSG00000153162.7
LYZL1	9.42201	ENSG00000120563.4
APIP	9.39819	ENSG00000149089.7
CD180	9.39819	ENSG00000134061.4
AP2B1	9.39206	ENSG00000006125.11
NAV2	9.39206	ENSG00000166833.14
SEPT11	9.39206	ENSG00000138758.7
SUB1	9.39206	ENSG00000113387.6
LTBP4	9.37013	ENSG00000090006.12
NANS	9.37013	ENSG00000095380.10
CCR7	9.34427	ENSG00000126353.2

ETS1	9.34427	ENSG00000134954.9
CLIC4	9.33599	ENSG00000169504.9
CSRP1	9.30942	ENSG00000159176.8
KRT8	9.28246	ENSG00000170421.6
RFPL4B	9.25912	ENSG00000251258.1
GSN	9.24765	ENSG00000148180.9
NDST1	9.24765	ENSG00000070614.9
PRKCD	9.24765	ENSG00000163932.9
UGP2	9.24765	ENSG00000169764.10
ATXN10	9.22719	ENSG00000130638.10
VEZT	9.19492	ENSG00000028203.12
GLS	9.11817	ENSG00000115419.8
TNC	9.10013	ENSG00000041982.9
BIRC2	9.00927	ENSG00000110330.3
PTEN	9.00927	ENSG00000171862.5
UMPS	9.00927	ENSG00000114491.9
SPINK4	9.00919	ENSG00000122711.4
CAT	8.99915	ENSG00000121691.4
TMEM99	8.99915	ENSG00000167920.4
C1QTNF6	8.95884	ENSG00000133466.9
COX4NB	8.95884	ENSG00000131148.3
GLRX	8.95884	ENSG00000173221.9
IRF1	8.95884	ENSG00000125347.8
RAB34	8.95884	ENSG00000109113.11
RFX2	8.95884	ENSG00000087903.7
RND1	8.95884	ENSG00000172602.5
RNF126	8.95884	ENSG00000070423.10
SDC4	8.95884	ENSG00000124145.5
SPAG9	8.95884	ENSG00000008294.14
ATG13	8.95726	ENSG00000175224.10
DPP3	8.95726	ENSG00000254986.2
EIF4A2	8.95726	ENSG00000156976.10
FAM111A	8.95726	ENSG00000166801.11
GPR137	8.95726	ENSG00000173264.8
GPR56	8.95726	ENSG00000205336.6
HLA-A	8.95726	ENSG00000206503.6
IL32	8.95726	ENSG00000008517.12
MAPK14	8.95726	ENSG00000112062.6
NBL1	8.95726	ENSG00000158747.9
NFKB2	8.95726	ENSG00000077150.12
SLC30A7	8.95726	ENSG00000162695.6
TRIM15	8.95726	ENSG00000204610.6
UPP1	8.95726	ENSG00000183696.9
WRAP53	8.95726	ENSG00000141499.10
ZBTB1	8.95726	ENSG00000126804.9
CCDC64	8.91188	ENSG00000135127.6

GFPT2	8.89717	ENSG00000131459.8
NOS1	8.89717	ENSG00000089250.13
C1orf213	8.86832	ENSG00000249087.3
HNF1A	8.86832	ENSG00000135100.9
IBTK	8.85921	ENSG00000005700.10
ALDH1B1	8.8582	ENSG00000137124.5
C7orf57	8.8582	ENSG00000164746.9
CCDC157	8.8582	ENSG00000187860.6
LEPROTL1	8.8582	ENSG00000104660.13
RRP1B	8.8582	ENSG00000160208.11
SH2B2	8.8582	ENSG00000160999.8
LRRN3	8.83502	ENSG00000173114.7
LIF	8.83225	ENSG00000128342.4
KLF14	8.80206	ENSG00000174595.4
LAMA4	8.80206	ENSG00000112769.12
LIG4	8.80206	ENSG00000174405.8
MYL10	8.80206	ENSG00000106436.4
ZFYVE26	8.80206	ENSG00000072121.10
CAPNS1	8.78439	ENSG00000126247.4
GSDMD	8.78439	ENSG00000104518.6
TRIP10	8.77081	ENSG00000125733.10
RFFL	8.7618	ENSG00000092871.11
ALX3	8.74503	ENSG00000156150.5
BTBD10	8.74503	ENSG00000148925.5
C3orf32	8.74503	ENSG00000125046.10
COX7A2L	8.74503	ENSG00000115944.9
DLGAP1	8.74503	ENSG00000170579.8
GSDMC	8.74503	ENSG00000147697.4
KCNH5	8.74503	ENSG00000140015.15
SDR42E1	8.74503	ENSG00000184860.4
METTL11A	8.74201	ENSG00000148335.8
C17orf80	8.73759	ENSG00000141219.10
C1orf31	8.73759	ENSG00000168275.9
C9orf91	8.73759	ENSG00000157693.10
IGFBP4	8.73759	ENSG00000141753.5
MEST	8.73759	ENSG00000106484.8
NAT10	8.73759	ENSG00000135372.4
RAD23B	8.73759	ENSG00000119318.7
RPLP1	8.73759	ENSG00000137818.7
C11orf86	8.72737	ENSG00000173237.4
LAMTOR2	8.72737	ENSG00000116586.7
NDUFV2	8.72737	ENSG00000178127.7
PPP1R15A	8.72737	ENSG00000087074.5
SEC24A	8.72737	ENSG00000113615.8

Supplementary Table 5. Ordered list of genes ordered by their closeness to MACS peaks. This is the naïve approach. These scores are MACS scores and are not comparable to the scored odds in Supplementary Table 4.

GeneName	Score	# of close peaks
REPIN1	2226.55	3
ALCAM	1439.73	2
ICAM1	1380.86	1
PLXNB2	1305.43	2
TNIP1	1285.59	2
ECT2	1270.78	1
S100A10	1188.13	2
MAP3K8	1149.15	1
TICAM1	1123.81	1
RHCG	1119.53	2
NXT2	1090.65	1
ADCK4	1065.41	1
NFKB2	989.05	1
RBM47	914.35	1
UXS1	905.16	2
KTN1	877.16	1
IFNAR2	849.93	1
PSEN1	822.8	1
CFLAR	822.72	2
ST5	814.87	2
RND1	790.83	1
FBXO46	784.4	1
NFKBIA	778.71	1
SYNPO	768.5	2
TNFRSF10B	754.07	1
CHDH	744.29	1
NR4A1	738.25	2
NNMT	737.63	1
RASA2	732.88	1
NFE2L2	731.35	3
C2	723.41	1
HAVCR1	723.37	1
IST1	715.12	1
CFB	714.23	1
SYT1	710.46	1
PPP1R15B	705.71	1
TRIM31	698.88	1
RARRES1	697.89	1
TRIM25	694.07	1
GALNTL4	688.4	1
PLEC	683	2
MSH5	679.38	1

CLIC1	679.38	1
ARL5B	676.97	1
SLC16A1	675.77	1
SQSTM1	672.59	1
C3	670.97	1
RFFL	667.93	2
MYH9	667.85	1
KLF4	662.03	1
TMBIM1	658.59	1
CXXC5	647.48	2
TANK	644.38	2
C3orf19	640.76	1
BIRC3	639.37	1
DENND4A	634.95	1
C12orf39	628.73	1
BIRC2	626.39	1
NFKBID	620.34	1
NFKBIB	613.28	1
DNAH7	609.29	1
MAFF	604.81	2
BCL3	591.85	1
PATL1	591.67	1
NFKBIZ	587.75	1
NCOA7	580.73	2
CREB1	579.59	2
HMGXB4	578.92	1
C12orf61	578.63	1
TP53I3	576.84	1
FAM129A	575.61	1
TBL1X	575.13	1
NEK6	571.15	2
PTMA	569.97	1
ARHGEF2	567.76	1
JMY	561.92	1
SH3BP4	560.8	1
ELF3	559.2	1
DIO2	558.46	1
EBI3	554.88	1
TMTC2	554.4	1
BFSP1	553.69	2
SRC	551.51	1
OLA1	550.66	2
PTGS2	549.62	1
PLCE1	548.72	1
KDM2A	540.86	1
THAP6	537.87	1

RCHY1	537.87	1
GSDMD	534.98	1
SMPD1	533.63	1
SH2D3A	533.48	1
CCDC107	532.79	1
FOSL2	532.62	1
PRRT2	530.82	1
IER5	523.36	1
BOD1	523.32	1
BHLHE40	518.41	1
TPRA1	518.28	1
STX11	515.98	1
IKBKB	513.13	1
CCDC57	512.31	1
PLLP	510.3	1
UNKL	508.68	1
BRE	505.54	1
RBKS	505.54	1
STAT6	503.58	1
CFL2	503.07	1
FOXK1	498.59	1
UBD	497.11	1
CCL20	494.45	1
DUSP3	493.72	1
UBQLN4	493.51	1
PDCD5	492.97	1
KLHL5	492.54	1
PLA2G4C	492.48	1
SDCBP	491.86	1
SLC9A8	491.6	1
PLEKHG2	488.63	1
CXCL1	486.47	1
PPAPDC2	484.24	1
WDR45L	483.81	1
PAN2	481.42	1
ATG13	480.91	1
APLF	479.8	1
C12orf59	478.24	1
CXCL2	477.94	1
MYADM	477.72	2
STARD3NL	477.38	1
ITGB2	474.62	1
MLL5	473.86	1
TSPAN15	469.99	1
S100A2	466.05	1
PDE4D	464.77	1

RPL23A	464.6	1
CDC42EP4	463.97	2
SLC7A2	460.62	1
ITSN2	460.3	1
PTTG1IP	457.24	1
C1S	456.05	1
CD34	455.97	1
TFPI2	454.44	1
RNF126	453.25	1
ETS2	451.6	1
SIAH1	451.55	1
VEZF1	451.19	1
IL4I1	450.89	1
MID1IP1	450.65	1
GPR56	449.46	1
PANX1	448.1	1
CPLX2	447.4	1
CD40	445.55	1
CLDN4	444.76	1
UBN2	443.53	1
MAPK14	441.19	1
BTG3	440.65	1
LGALS1	440.46	1
PDZD2	440.23	1
GPX4	439.57	1
C11orf46	438.78	1
TMEM105	438.55	1
C1orf213	437.41	1
ZNF436	437.41	1
TESK2	434.13	1
TNFSF15	432.32	1
CCNL1	431.56	1
BTBD19	430.96	1
C10orf11	430.92	1
RNF19A	430.03	2
GCH1	429.83	1
NEO1	428.55	1
MAFG	428.5	1
SLC30A7	426.29	1
TXNRD1	425.78	1
SLC11A2	424.08	1
UPP1	423.65	1
PFN1	423.35	1
ENO3	423.35	1
ABHD2	421.72	1
TRIM44	421.7	1

CYBA	421.08	1
NR4A2	420.37	2
CCRN4L	419.76	1
SENP3	419.7	1
CLTA	416.08	1
BBC3	414.47	1
FOSL1	414.18	1
MAP3K11	413.2	1
RPS27A	412.1	1
ESPL1	410.22	1
SLC7A5	408.44	1
QKI	406.95	1
SYNGR2	406.2	1
CD55	404.92	1
TP63	403.77	1
C1orf116	400.79	1
GADD45B	399.32	1
DUSP10	398.52	1
RNF31	398.49	1
PSME2	398.49	1
SLC39A10	398.29	1
AP1S3	397.03	1
ZNF574	396.58	1
ASAP1	396.54	1
CD63	395.51	1
ARL6IP5	394.29	1
DDX26B	393.99	1
CACHD1	393.6	1
AP2B1	390.92	1
SKIL	390.77	1
CDK12	390.65	1
CAV1	390.28	1
CARD14	389.04	1
ATXN2L	388.71	1
INHBE	387.2	1
LENG9	386	1
ZFP36L1	384.69	1
LAMB3	383.01	1
TLL1	382.77	1
MUC1	381.56	1
ZHX2	381.4	1
DHRS7B	380.51	1
UQCRC2	377.68	1
THOC1	376.66	1
PPP6R3	376.39	1
MMD	376.2	1

PAWR	374.87	1
ZFHX3	374.54	1
SSSCA1	374.38	1
DIAPH2	374.28	1
RFX5	373.13	1
STRA6	372.9	1
IFT20	372.85	1
TNFAIP1	372.85	1
CD59	371.4	1
SLC5A2	371.33	1
PPIF	370.77	1
MED13	369.3	1
PTK6	367.11	1
NOD2	366.83	1
RAB27A	365.51	1
TRIM15	365.02	1
SLC25A45	364.55	1
FAM111A	363.37	1
TLE1	363.31	1
EPS8	363.21	1
MUC2	362.89	1
NINJ1	360.9	1
PI3	360.88	1
SYS1	360.46	1
RASSF9	359.43	1
NEK8	356.64	1
SLC48A1	356.64	1
RAPGEF3	356.64	1
RCAN1	356.61	1
NBL1	355.64	1
AP2A1	354.51	1
NDUFV2	354.42	1
IER3	354.36	1
CYB5A	354.28	1
ERF	354.14	1
C2orf15	353.46	1
MITD1	353.46	1
CYTH1	352.8	1
GCNT3	351.3	1
TRIM47	351.23	1
PDE6D	350.98	1
STX4	350.97	1
BNIP1	350.39	1
PARK7	349.24	1
GTF2E1	347.24	1
B3GAT3	346.3	1

SOX13	345.95	1
TMEM102	345.59	1
MRPS24	344.59	1
CLCF1	342.8	1
TAF4B	342.07	1
NUMB	341.15	1
CTSS	340.34	1
AZIN1	339.75	1
AKR1B1	339.06	1
SLC31A1	338.14	1
FKBP15	338.14	1
PDE10A	337.74	1
HNF4A	336.93	1
WDR16	334.99	1
STX8	334.99	1
USP49	334.93	1
WDR7	334.41	1
TXNL1	334.41	1
DLG4	333.63	1
TSPAN14	333.36	1
ASB9	333.3	1
CXCL3	332.82	1
ANKLE2	332.67	1
RAB3IP	332.23	1
RHOG	332.1	1
TRIP4	331.75	1
MAML2	331.21	1
IL32	331.08	1
ITFG3	330.84	1
ZNF101	330.79	1
NFATC2IP	330.45	1
NBEAL1	330.18	1
C9orf142	329.27	1
TAPBP	326.87	1
TBL1XR1	326.77	1
CDC14B	326.6	1
NDE1	326.11	1
KIAA0430	326.11	1
DIRC2	325.7	1
CBX5	324.03	1
C16orf46	323.77	1
LSR	323.57	1
MARCKS	323.23	1
AMOTL2	322.59	1
CSF1	321.82	1
MASTL	321.54	1

ATG16L2	321.35	1
DSTN	321.24	1
STX1A	321.09	1
PRMT1	320.88	1
CYB561	320.68	1
PRKCD	320.54	1
SLPI	319.93	1
ITGB8	319.19	1
MYO1G	319.03	1
APOBEC3A	318.94	1
MKLN1	318.41	1
TSG101	317.3	1
IRF2BP2	316.75	1
SMG1	315.85	1
CCDC64	315.53	1
SNCAIP	313.46	1
EFEMP2	312.46	1
ST3GAL1	311.37	1
IFIH1	311.19	1
FSTL3	309.77	1
SP3	309.27	1
UBB	309.1	1
LBR	309.07	1
AC092811.1	309.07	1
C11orf80	308.44	1
GRB7	308.04	1
DDX47	307.12	1
PTP4A2	306.66	1
ANKRD18B	306.38	1
PTPN12	306.1	1
CCDC124	305.91	1
WWC1	305.84	1
CBR3	305.3	1
PABPC1	305.17	1
DTX4	305	1
IGSF3	304.55	1
ART4	304.21	1
MAP2K3	303.88	1
NTRK3	303.34	1
GATSL3	303.03	1
AGR2	302.99	1
TFE3	302.81	1
PTGIR	302.56	1
STK40	302.34	1
GRN	301.52	1
LARP6	301.15	1

PTGES	301.09	1
CCDC97	298.93	1
IGF2BP1	298.19	1
SYT2	297.54	1
ACSS1	297.16	1
NFIC	296.97	1
CS	296.65	1
IRF1	296.64	1
DUSP6	296.34	1
ALPK1	294.95	1
ESCO2	294.66	1
NPRL2	294.57	1
FCHSD2	294.21	1
TRAPPC10	293.73	1
RBM17	292.94	1
ALDH3A1	292.43	1
UGT1A6	292.21	1
KIF26A	292.18	1
C15orf52	291.46	1
UPF3B	290.26	1
PCDH1	289.62	1
ANXA7	289.49	1
ADRBK2	289.33	1
COTL1	289.32	1
AKAP13	289.21	1
DICER1	288.42	1
INO80C	288.37	1
FFAR2	287.74	1
CLIC4	287.73	1
PARD3	287.68	1
SLC24A6	287.52	1
PHLPP2	287.17	1
ASB6	287.1	1
ARNT2	286.52	1
PLAU	285.95	1
CCDC90A	285.94	1
TPBG	285.85	1
TNFSF13	285.52	1
NUB1	285.14	1
ZNF33B	284.85	1
FCHO1	284.54	1
ZFP91	284.39	1
LPXN	284.39	1
HES1	283.58	1
RBM14	282.93	1
C11orf86	282.6	1

ATP9A	281.17	1
LZIC	281.14	1
NMNAT1	281.14	1
RBM23	280.41	1
CD70	279.92	1
P2RY6	279.43	1
STAT5A	278.54	1
CTSB	278.49	1
11-Sep	278.12	1
LCN2	278.08	1
SLC50A1	277.77	1
PHLDA1	277.66	1
ZDHHC24	277.55	1
HELLS	277.34	1
GPR137	277.21	1
SLITRK5	276.41	1
SERINC4	275.52	1
C15orf63	275.52	1
TRIOBP	274.86	1
NRXN2	274.37	1
C12orf34	274.3	1
EIF2AK3	273.17	1
KANSL3	272.99	1
SRCIN1	272.87	1
GRAMD1A	272.86	1
RUSC1	272.8	1
FARS2	272.6	1
LYRM4	272.6	1
ZFHX2	272.33	1
PRKCH	272.26	1
DPP3	271.85	1
CDC37	271.75	1
SHC1	271.53	1
CKS1B	271.53	1
CYFIP1	271.46	1
CSRP2	271.41	1
VPS53	271.27	1
CCDC115	271.2	1
WDR31	270.73	1
BCAR3	270.43	1
DOCK10	270.18	1
WDR11	269.91	1
DUSP5	269.51	1
C1orf132	269.36	1
PRKAB2	268.87	1
ARL14	267.74	1

ASS1	267.68	1
PCMTD1	267.48	1
ZNF33A	266.65	1
NXT1	266.6	1
CANT1	266.08	1
ZNF823	265.64	1
NR1D1	265.58	1
IKBKE	265.36	1
GNAL	265.32	1
BLOC1S1	265.12	1
PFDN4	264.45	1
ARHGEF40	264.24	1
NDST1	263.89	1
EHD2	263.78	1
DCLRE1A	263.61	1
NHLRC2	263.61	1
SRGAP1	263.27	1
UMPS	263.18	1
ZNF706	263.03	1
PIK3C2B	263.03	1
FLNA	262.97	1
RPS2	262.86	1
PARP1	262.84	1
C19orf21	262.63	1
SUB1	262.12	1
MGLL	261.95	1
SPECC1	261.93	1
AJUBA	261.58	1
SPATA2L	261.24	1
SLC25A37	260.81	1
TBC1D10A	260.67	1
HLA-A	260.11	1
FLOT2	259.89	1
CXorf36	259.83	1
IL18R1	258.26	1
PBX2	258.16	1
ITPRIP	257.85	1
SIX5	257.15	1
CALD1	256.86	1
GPBP1	256.71	1
MUC17	256.65	1
SS18	256.26	1
IL8	256.18	1
MID2	256.03	1
WDR89	256.01	1
SLC25A12	255.97	1

TMEM75	255.95	1
COX4I1	255.08	1
COX4NB	255.08	1
LTBP4	255.02	1
RPH3AL	254.83	1
SERPINB8	254.59	1
SUPT16H	254.33	1
GABPB1	254.32	1
GLRX	253.73	1
SPPL2A	252.91	1
AGTRAP	252.79	1
TRIM69	251.7	1
ZBTB1	251.64	1
ZBTB25	251.64	1
SF3B1	251.23	1
DHPS	250.95	1
ACHE	250.71	1
C3orf17	250.44	1
PDCD2L	249.9	1
TCF7L2	249.81	1
HNRNPF	249.79	1
CYP3A5	249.06	1
SRRT	248.35	1
FECH	248.22	1
SRCAP	247.85	1
NFAT5	247.32	1
SLC36A4	247.06	1
RUFY1	247.02	1
C20orf24	246.99	1
PRKAA1	246.97	1
GPRC5A	246.95	1
H3F3B	246.94	1
HSPA1L	245.63	1
HSPA1A	245.63	1
STK39	245.62	1
PHLDB1	245.39	1
TFF1	245.06	1
AP4M1	244.61	1
MCM7	244.61	1
CLDN16	244.59	1
GFRA1	243.98	1
ZNF467	243.54	1
TAF1B	243.05	1
ENO1	242.49	1
TBRG4	242.1	1
FAM82B	241.77	1

DPP4	241.29	1
PJA2	241.12	1
ZC2HC1C	240.9	1
NFKB1	240.85	1
NANS	240.51	1
HMGCS1	240.11	1
NAMPT	239.58	1
SMCHD1	238.63	1
GALT	238.27	1
TMPRSS9	238.11	1
HMSD	237.57	1
PARP4	237.57	1
KCNT2	237.23	1
ZFP161	237.12	1
ZCCHC8	236.38	1
IL1A	236.37	1
BAIAP2L2	236.24	1
PER1	236.08	1
SDC4	235.9	1
LINC00346	235.17	1
UGP2	235	1
TINAG	234.8	1
UNC13D	234.76	1
RAB30	234.68	1
EGR1	234.18	1
SNUPN	234.12	1
EIF4A2	234.1	1
C1orf86	233.66	1
NOTCH2NL	233.54	1
PNKD	233.37	1
C14orf43	233.36	1
S100A3	232.98	1
S100A4	232.98	1
PPP1R14B	232.95	1
ZNF799	232.72	1
TRAF4	232.69	1
SLC3A2	232.55	1
ARID1A	232.36	1
ZNRF1	231.91	1
SNAPC1	231.8	1
PTGER4	231.46	1
CHST15	231.18	1
CXCL5	231.09	1
GPR108	230.89	1
TEF	230.79	1
CHD2	230.45	1

GEM	230.12	1
LRRIQ1	229.48	1
BCAR1	229.41	1
PRRG4	229.01	1
C7orf23	228.64	1
MST1P9	228.28	1
ODZ3	227.87	1
RELA	227.83	1
CXCL10	227.57	1
PHLDB2	227.05	1
F3	226.68	1
ADCK3	226.25	1
METTL2B	225.73	1
CCDC94	225.7	1
GSN	225.51	1
GGT1	225.5	1
TCF19	225.49	1
PRCP	225.37	1
NDUFB7	225.03	1
UGT2A3	224.78	1
NEU1	224.77	1
C11orf54	224.59	1
C1orf63	224.59	1
TMEM50A	224.59	1
TAF1D	224.59	1
RB1CC1	224.45	1
TRAPPC3	224.44	1
ERI2	224.31	1
PWWP2A	224.27	1
CAPNS1	224.14	1
SLC29A1	224.09	1
STAT1	223.86	1
PLEKHG6	223.85	1
HNF1A	223.55	1
TTC23L	223.31	1
RAB11FIP5	222.79	1
EIF2C2	222.65	1
MTMR2	222.09	1
DUSP22	221.91	1
FBXW11	221.44	1
KIAA1609	221.31	1
IL17F	221.12	1
RFX2	221.02	1
SAR1B	220.84	1
SEC24A	220.84	1
COLEC10	220.51	1

TFG	220.38	1
CASP10	220.27	1
BCAT1	219.89	1
GSTP1	219.81	1
TLCD1	219.5	1
JUN	219.46	1
CA13	219.41	1
G3BP1	219.25	1
ATOX1	219.25	1
DYRK2	219.23	1
TMBIM4	218.73	1
SULT1C4	218.55	1
PRPF39	218.5	1
NAV1	217.88	1
KLHL21	217.56	1
RILPL2	217.43	1
GLI1	217.26	1
VRK1	217.08	1
MLLT11	217.05	1
MID1	216.76	1
CDK17	216.51	1
IKZF3	216.46	1
TWISTNB	216.44	1
ARHGAP27	216.28	1
ZBED3	216.12	1
NDUFA7	215.85	1
RPS28	215.85	1
HILPDA	215.69	1
NCOA4	215.67	1
MED15	215.54	1
SPAG9	215.37	1
TSKU	214.84	1
ITGB1	214.61	1
BDKRB2	214.6	1
ZBBX	214.49	1
LPGAT1	214.37	1
PAPPA	213.83	1
C1QTNF6	213.77	1
ARL4A	213.64	1
PPIL6	213.62	1
SMPD2	213.62	1
PTEN	213.46	1
KLLN	213.46	1
ZBTB10	212.44	1
SEMA4C	212.44	1
PDGFRA	211.82	1

RIPK2	211.81	1
FOXP4	211.71	1
VHL	211.28	1
ABI1	211.23	1
CDC42EP5	210.73	1
FASTKD5	210.51	1
UBOX5	210.51	1
POLR2A	210.23	1
NAV2	210.01	1
FAM103A1	209.9	1
C8orf37	209.88	1
TBC1D12	209.86	1
MAP7	209.62	1
TOM1	209.56	1
TIMM17B	209.07	1
PQBP1	209.07	1
DENND3	208.75	1
BIN3	208.49	1
DCTPP1	208.49	1
S100A13	208.33	1
CHTOP	208.33	1
SIX4	208.26	1
PPP1R15A	208.2	1
KLK10	208.05	1
IZUMO1	208.05	1
TPT1	207.74	1
RAPH1	207.71	1
KCNS3	207.52	1
PCSK2	207.19	1
WRAP53	206.99	1
TP53	206.99	1
WWP1	206.72	1
SLC52A3	206.6	1
TPCN1	205.62	1
PRRC2C	205.61	1
LMNA	205.45	1
USP54	205.25	1
NIPA1	204.96	1
SLC16A13	204.93	1
GRK5	204.42	1
DNAJB6	203.86	1
RARG	202.83	1
ZFX	202.82	1
PTHLH	202.61	1
WBP1	202.57	1
PPTC7	201.98	1

LRG1	201.8	1
FAM83G	201.53	1
DDR1	201.31	1
ST7	201.3	1
EHBP1	201.26	1
SELE	201.16	1
ZNF438	201.11	1
MLLT6	201.09	1
KCNMA1	200.96	1
FER1L6	200.78	1
C4orf19	200.76	1
CDH23	200.66	1
B4GALT5	200.46	1
BRI3BP	200.44	1
SHC3	200.32	1
RAB21	200.2	1
CSAD	200.14	1
FSCN1	200.1	1
TPD52	200.08	1
